# miRNA-148a–containing GMSC-derived EVs modulate Treg/Th17 balance via IKKB/NF-**κ**B pathway and treat a rheumatoid arthritis model

**DOI:** 10.1172/jci.insight.177841

**Published:** 2024-04-23

**Authors:** Jingrong Chen, Xiaoyi Shi, Yanan Deng, Junlong Dang, Yan Liu, Jun Zhao, Rongzhen Liang, Donglan Zeng, Wenbin Wu, Yiding Xiong, Jia Yuan, Ye Chen, Julie Wang, Weidong Lin, Xiangfang Chen, Weishan Huang, Nancy Olsen, Yunfeng Pan, Qingling Fu, Song Guo Zheng

**Affiliations:** 1 Department of Immunology, School of Cell and Gene Therapy, Shanghai Songjiang Hospital Affiliated to Shanghai Jiao Tong University School of Medicine, Shanghai, China.; 2 Department of Internal Medicine, Division of Rheumatology, The Third Affiliated Hospital of Sun Yat-sen University, Guangzhou, China.; 3 Department of Transplantation, Nanfang Hospital, Southern Medical University, Guangzhou, China.; 4 Department of Clinical Immunology,; 5 Department of Spine Surgery, and; 6 Department of Stomatology, The Third Affiliated Hospital of Sun Yat-sen University, Guangzhou, China.; 7 Department of Endocrinology, Second Affiliated Hospital of Naval Medical University, Shanghai, China.; 8 Department of Pathobiological Sciences, School of Veterinary Medicine, Louisiana State University, Baton Rouge, Louisiana, USA.; 9 Division of Rheumatology, Department of Medicine, The Penn State University Hershey Medical Center, Hershey, Pennsylvania, USA.; 10 Otorhinolaryngology Hospital, The First Affiliated Hospital, Sun Yat-sen University, Guangzhou, China.

**Keywords:** Autoimmunity, Stem cells, Autoimmune diseases, Stem cell transplantation

## Abstract

Mesenchymal stem cells (MSCs) have demonstrated potent immunomodulatory properties that have shown promise in the treatment of autoimmune diseases, including rheumatoid arthritis (RA). However, the inherent heterogeneity of MSCs triggered conflicting therapeutic outcomes, raising safety concerns and limiting their clinical application. This study aimed to investigate the potential of extracellular vesicles derived from human gingival mesenchymal stem cells (GMSC-EVs) as a therapeutic strategy for RA. Through in vivo experiments using an experimental RA model, our results demonstrate that GMSC-EVs selectively homed to inflamed joints and recovered Treg and Th17 cell balance, resulting in the reduction of arthritis progression. Our investigations also uncovered miR-148a-3p as a critical contributor to the Treg/Th17 balance modulation via IKKB/NF-κB signaling orchestrated by GMSC-EVs, which was subsequently validated in a model of human xenograft versus host disease (xGvHD). Furthermore, we successfully developed a humanized animal model by utilizing synovial fibroblasts obtained from patients with RA (RASFs). We found that GMSC-EVs impeded the invasiveness of RASFs and minimized cartilage destruction, indicating their potential therapeutic efficacy in the context of patients with RA. Overall, the unique characteristics — including reduced immunogenicity, simplified administration, and inherent ability to target inflamed tissues — position GMSC-EVs as a viable alternative for RA and other autoimmune diseases.

## Abstract 2

Extracellular vesicles derived from human gingival mesenchymal stem cells traffic to inflamed joints and halt cartilage destruction in a mouse model of rheumatoid arthritis.

## Graphical abstract

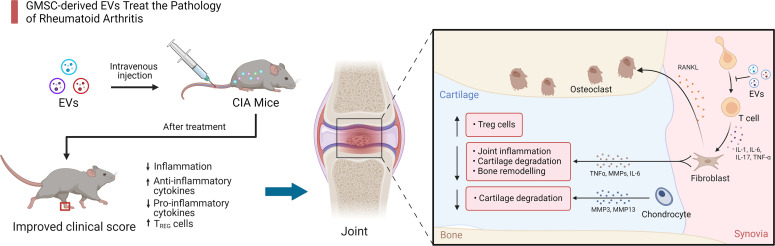

## Introduction

Rheumatoid arthritis (RA) is a common autoimmune disease characterized by persistent joint inflammation and destruction of cartilage and bone (1, 2). An increasing amount of evidence indicates that mesenchymal stem cells (MSCs) have the potential to fight against autoimmune and inflammatory diseases, including autoimmune arthritis (3–10). However, several concerns arise in clinical practice. For example, MSC in patients are usually dysfunctional, making allogenic MSC transfer the only option, which may trigger immune rejection. Moreover, the long-term cell fate of the transferred MSC in patients remains largely unclear; there are also common side effects, including cellular toxicity and tumorigenesis (11–13). An effective immune therapy depends on precise targeting and potent immune modulation. Current RA treatment regimens involving immune suppressants often require high doses of drugs to show a therapeutic effect in the affected joints, doses which often trigger adverse off-target effects on normal tissues. The current cell-based therapeutic strategies against inflammation often lack homing specificity to the inflamed sites, and this limits their applications in the clinic. Developing innovative therapeutic approaches that are devoid of cells and specifically target RA is of utmost importance.

Recent studies identified that many cells exert their function through extracellular vesicles (EVs). There are 2 main categories of EVs, namely ectosomes and exosomes (14, 15). Ectosomes, which consist of microvesicles, microparticles, and large vesicles ranging from approximately 50 nm to 1 μm in diameter, are formed by outward budding and separate from the plasma membrane. Exosomes, which have a size ranging from 30 to 160 nm, are discharged into the extracellular matrix when the fusion of multivesicular bodies (MVB) with the plasma membrane occurs (14, 16, 17). Since there is no agreement yet on distinct indicators of EV subcategories, it becomes challenging to differentiate between exosomes or microvesicles. Hence, exosomes or microvesicles are commonly denoted as small EVs, in accordance with the classical references (18–20). According to reports, EVs may facilitate the paracrine effects of MSCs, enhance tissue healing and immune suppression, and uphold homeostasis (21).

In our current study, we reveal discoveries that demonstrate the effectiveness of EVs derived from human GMSCs (GMSC-EVs) in treating an animal model of RA. Significantly, miR-148a has been recognized as a noteworthy participant in GMSC-EVs, exerting a crucial influence on the suppression of immune response and the reduction of disease progression by specifically modulating the IKKB/NF-κB signaling pathway. Our research highlights the vast possibilities of GMSC-EVs as an innovative and hopeful treatment without cells to fight against RA as well as various other autoimmune disorders.

## Results

### Human GMSC–derived EVs suppress T cell activation, proliferation, differentiation, and inflammatory cytokine production in vitro.

**Figure 1:**
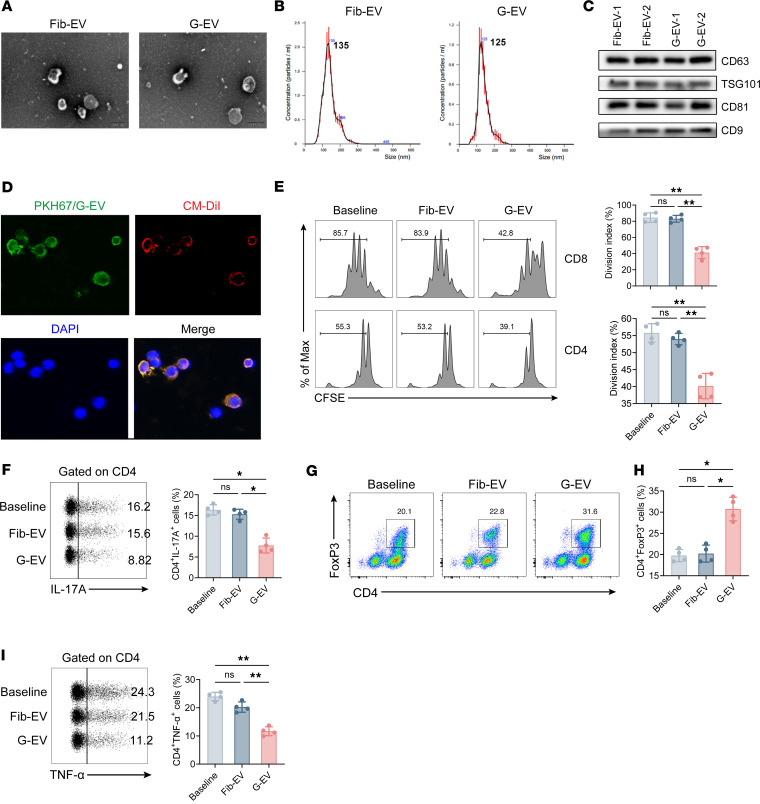
Human GMSC–derived EVs inhibit T cell responses in vitro. (**A**) Electron micrograph analysis of the morphology of EVs. Scale bar: 200 nm. (**B**) Nanoparticle trafficking analyzed the diameters and concentration of EVs. (**C**) The EVs’ protein markers were detected by Western blot. (**D**) PKH67-labeled (green) GMSC-EVs were cocultured with CD3^+^ T cells under stimulation of soluble anti-CD3 and soluble anti-CD28 Abs after 1 days. Cells were harvested and stained with CM-DiI (red) and DAPI (blue), and then images were acquired by fluorescence confocal. (**E**) In vitro suppressive assay of T cell proliferation. (**F**) Th17-polarizing analysis. (**G** and **H**) Treg-polarizing analysis. (**I**) In vitro suppressive assay of cytokine production. Statistical significance was assessed by 1-way ANOVA with Dunnett multiple-comparison test in **E**–**I**. Data are shown as the means ± SD from 1 of 3 independent experiments. *P < 0.05; **P < 0.01.

GMSCs were analyzed using flow cytometry to investigate the cell surface markers. The findings of our study reveal that GMSCs exhibited the typical traits of MSCs (Supplemental Figure 1A; supplemental material available online with this article; https://doi.org/10.1172/jci.insight.177841DS1). Differential ultracentrifugation is widely adopted for EVs isolation from biological fluids and is, therefore, considered the “gold standard protocol” of EVs isolation (22, 23). Consequently, the GMSC-EVs (i.e., G-EVs) were successfully obtained and utilized for subsequent experiments (Figure 1, A–C, and Supplemental Figure 1B).

To ensure that T cells cultured with GMSC-EVs were not affected by cell apoptosis or death-induced nonspecific reactions, Annexin-V and PI staining was performed. The results demonstrate no overt side effects triggered by GMSC-EVs (Supplemental Figure 2, A and B). Furthermore, we examined the interactions between GMSC-EVs and T lymphocytes in a controlled environment and their capability to modulate the proliferation, differentiation, and activity of T cells. The results show that GMSC-EVs localized in the cytoplasmic compartment of T cells, indicating their uptake by T cells (Figure 1D). To learn whether GMSC-EVs suppress T cell activation, we examined the expression of the early activation marker CD69 on the T cells. The results demonstrate that GMSC-EVs significantly reduced the proportion of CD69^+^ cells in both CD4^+^ and CD8^+^ T cell populations, suggesting that GMSC-EVs start modulating T cell immune responses since T cells are initially primed (Supplemental Figure 3, A and B). In addition, the findings indicate that GMSC-EVs displayed strong inhibitory effects on the proliferation of CD8^+^ and CD4^+^ T cells, as demonstrated by decreased divisions observed through CFSE dilution (Figure 1E). Furthermore, the study found that GMSC-EVs, rather than fibroblast-derived EVs (Fib-EVs), had a significant inhibitory effect on the differentiation of Th17 (CD4^+^IL-17A^+^) cells (Figure 1F).

MSC-EVs have been demonstrated to affect the development of Tregs in a manner that depends on the donor, as indicated by previous studies (24, 25). The research findings indicate that the administration of GMSC-EVs improved the development of FoxP3^+^ Tregs when naive CD4 cells were stimulated under conditions that promote Treg polarization (Figure 1, G and H). Additionally, the quantities of inflammatory and noninflammatory cytokines can function as markers of immune balance. We observed that coculturing CD3^+^ T cells with GMSC-EVs significantly reduced the amounts of TNF-α by CD4^+^ T cells (Figure 1I). To summarize, our findings indicate that GMSC-EVs hindered the activation, growth, and differentiation of T cells, and they suppressed the production of proinflammatory cytokines while facilitating the development of Tregs.

### Human GMSC–derived EVs improve the collagen-induced arthritis (CIA) model.

**Figure 2:**
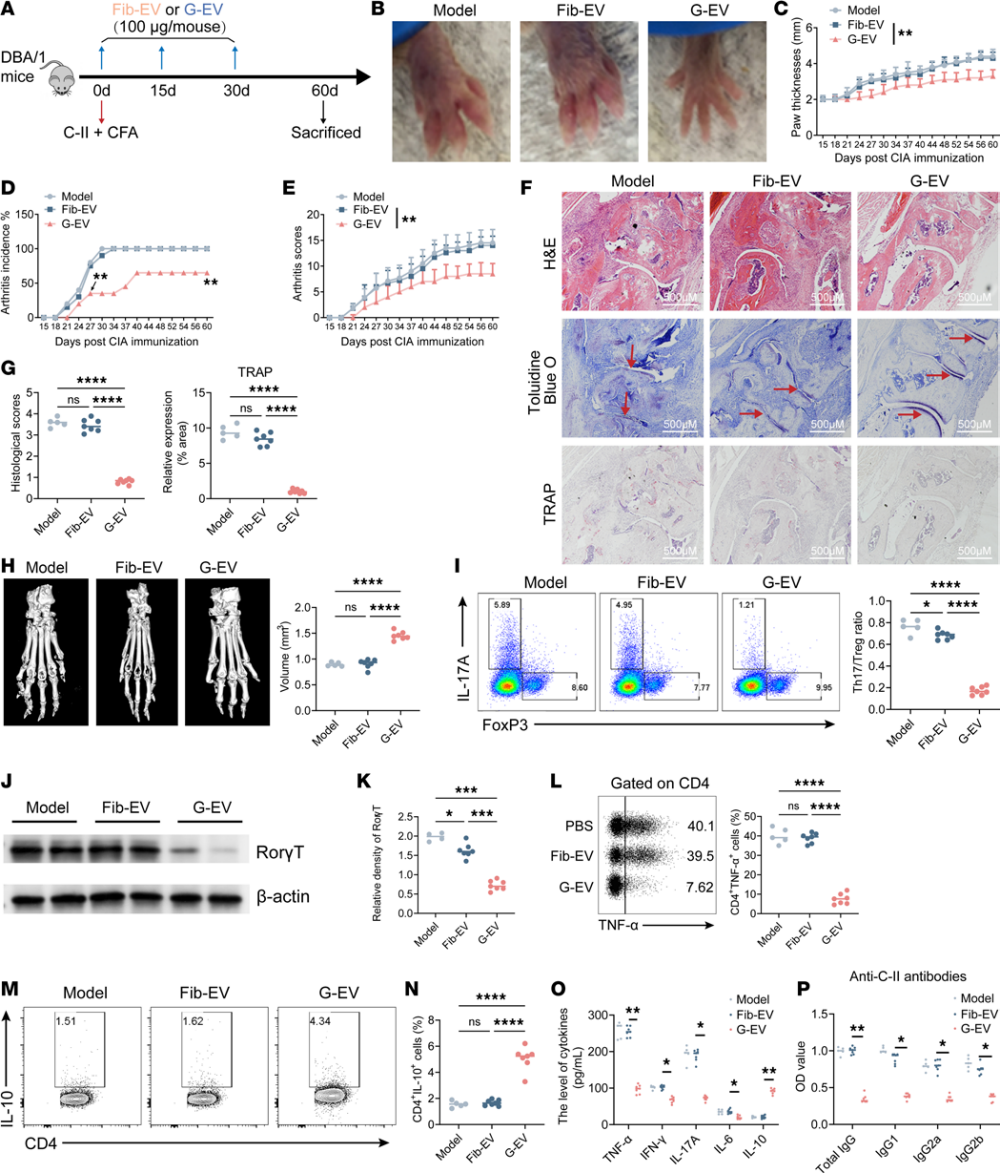
Human GMSC–derived EVs protect against collagen-induced arthritis (CIA) model. (**A**) Schematic diagram summarized the CIA modeling and G-EV administration. (**B**) The representative images of gross appearance of swollen hind paws at the endpoint of the experiment. (**C**–**E**) The paw thickness (**C**), incidence of arthritis (**D**), and arthritis severity scores (**E**) of CIA mice were monitored from day 15 to day 60 after immunization. (**F** and **G**) Ankle joint sections isolated from CIA mice at day 60 after immunization were stained with H&E and toluidine blue staining. Histopathologic scores were evaluated for features of synovitis, pannus, erosion, and cartilage matrix. The red arrows indicated the cartilage destruction of joints. Osteoclast distribution was quantified by tartrate acid resistant phosphatase (TRAP) staining. Scale bars = 500 μm. (**H**) Toe joint sections isolated from CIA mice at day 60 after immunization were imaged with μ-CT, and the structural damage was evaluated as bone volumes of the metatarsophalangeal joint indicated. (**I**) dLN cells isolated from CIA mice at day 60 after immunization for intracellular staining of IL-17A and Foxp3 by flow cytometry analysis. (**J** and **K**) Splenic cells isolated from CIA mice at day 60 after immunization were collected for the detection of the protein level of RORγt by Western blot analysis. (**L**–**N**) dLNs isolated from CIA mice at day 60 after immunization for intracellular staining of TNF-α and IL-10 in CD4^+^ cells by flow cytometry analysis. (**O** and **P**) Serum samples obtained from blood of CIA mice at day 60 after immunization were used for the detection of cytokines (**O**) and autoantibodies (**P**) by ELISA. Statistical significance was assessed by 1-way ANOVA with Dunnett multiple-comparison test in **C**–**P**. Data are mean ± SD, *n* = 5–8 mice. *P < 0.05; **P < 0.01; ****P < 0.0001.

In our prior investigation, we documented that GMSC greatly improved the pathology and inflammatory reactions in a mouse model of CIA (26). The pathological characteristics of human RA, such as synovial hyperplasia, joint swelling, and damage to bone and cartilage, are largely replicated in this experimental model (27, 28). To extend the prevention potential of GMSC-EVs on inflammatory arthritis, GMSC-EVs were administered to mice at different time points after immunization (Figure 2A). On day 60 after immunization, the gross appearance of hind limbs had a significant remission of arthritis in GMSC-EVs treatment mice versus that in disease model or Fib-EVs treatment mice (Figure 2B). The consistent foot swelling was noticed and monitored from day 15 to day 60, as indicated in Figure 2C. Moreover, the administration of GMSC-EVs resulted in a postponement of the initiation of arthritic ailment, a decrease in the occurrence of arthritis (Figure 2D), and a reduction in arthritis clinical scores (Figure 2E). Histological analysis revealed that GMSC-EV treatment resulted in decreased synovial hyperplasia, cartilage damage, and osteoclast activity (Figure 2, F and G). In order to assess the level of bone damage in CIA mice, we performed μ-CT scanning and observed a notable safeguarding impact on bone erosion in mice administered with GMSC-EVs (Figure 2H). These results indicate that GMSC-EVs have sufficient therapeutic potency on CIA mice.

Various pieces of evidence indicate that maintaining a proper equilibrium between Th17 cells, which produce IL-17A and promote inflammation, and Tregs, which are FoxP3^+^ and inhibit inflammation, is vital in autoimmune arthritis (29, 30). Our research reveals that treatment with GMSC-EVs led to a notable decrease in the occurrence of Th17 cells and a notable increase in the occurrence of Tregs in the draining lymph nodes (dLNs) (Figure 2I). Additionally, the expression and activity of RORγt, a transcription factor involved in Th17 cell development, were consistently inhibited in GMSC-EV–treated mice (Figure 2, J and K). The administration of GMSC-EVs significantly reduced the synthesis of TNF-α by CD4^+^ cells (Figure 2L) while simultaneously enhancing the release of IL-10, a cytokine known for its antiinflammatory properties (Figure 2, M and N). In addition, GMSC-EV treatment effectively reduced the levels of proinflammatory cytokines TNF-α, IFN-γ, IL-17A, and IL-6 in the blood, while simultaneously increasing the level of the antiinflammatory cytokine IL-10 (Figure 2O). We found that the introduction of GMSC-EVs also led to a decrease in the concentrations of autoantibodies in the blood samples (Figure 2P). Collectively, these findings indicate that GMSC-EVs have the ability to improve the pathology and reduce inflammatory responses in a model of inflammatory arthritis.

### The distribution of human GMSC–derived EVs in CIA model.

**Figure 3:**
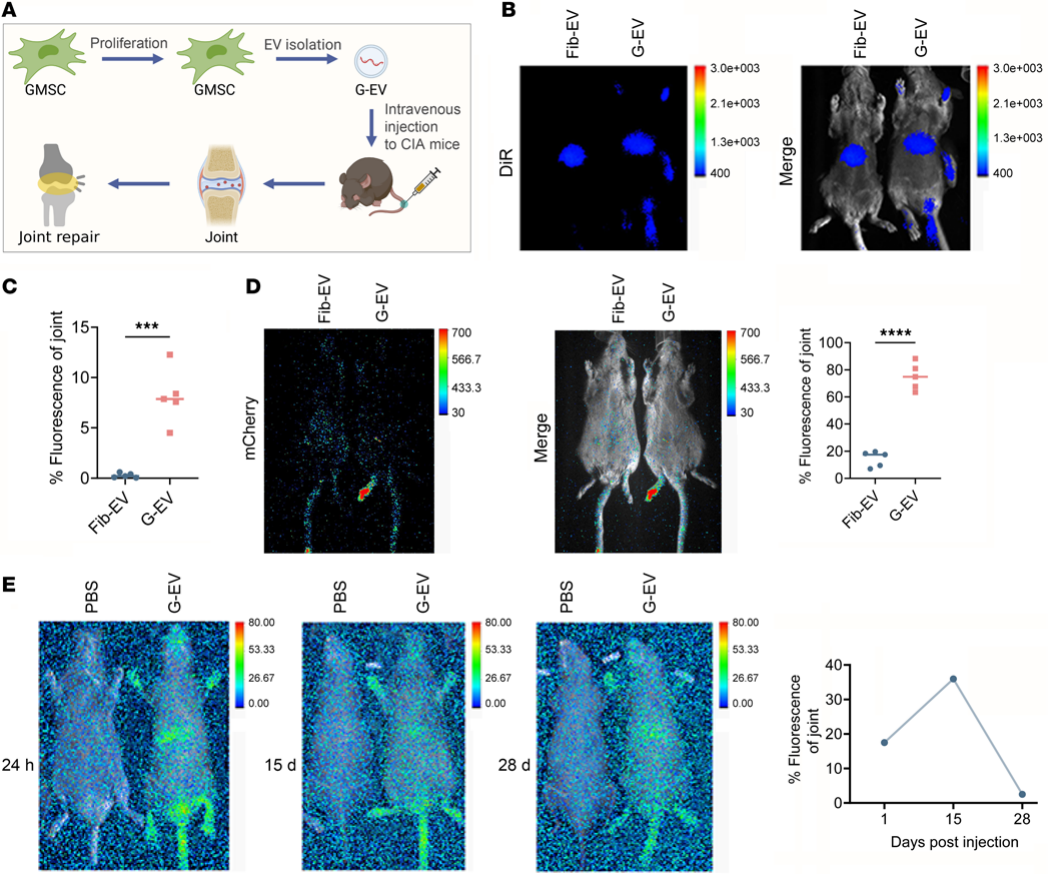
In vivo tracking of human GMSC–derived EVs in CIA mice. (**A**) Schematic illustration depicting the delivery of EVs to the joint via the tail vein for the treatment of CIA. (**B**) Twenty-four–hour following the administration of DiR-labeled (red) EVs in CIA mice 2, digital photo, and IVIS images were used to present the fluorescence signal. (**C**) Quantification of fluorescence percentage of joint in total for **B**. (**D**) In vivo imaging of mCherry-carried (red) EVs in CIA mice 24 hours after injection, and quantification of fluorescence percentage of joint in total. (**E**) In vivo imaging of DiR-labeled GMSC-EVs in CIA mice at 24 hours, 14 days, and 28 days after injection, and quantification of fluorescence percentage of joint in total. Left mouse received PBS as the control. Statistical significance was assessed with 2-tailed Student *t* test in **C** and **D**. Representative images from 3 separate experiments. ***P < 0.001; ****P < 0.0001.

In order to precisely determine the anatomical location of transferred GMSC-EVs in the CIA model, we conducted live imaging to analyze the dynamic distribution of GMSC-EVs throughout the entire animal body (Figure 3A). In this study, GMSC-EVs and Fib-EVs were labeled with a lipophilic tracer DiR prior to i.v. injection into CIA mice, and whole body images were obtained 24 hours later. Results indicate that GMSC-EVs homed preferentially to the inflamed joints, whereas Fib-EVs did not (Figure 3, B and C). However, concerns have been raised about the accuracy of using lipophilic dye staining for EV labeling due to, for example, potential nonspecific staining of other lipid-containing entities in the extracellular space, formation of dye aggregates or clumps, or different metabolism profiles from EVs. To address these issues, mCherry was fused to the COOH-termini of GFP for EV membrane labeling in our current study, using a CD63-mCherry-GFP lentivirus as an alternative labeling strategy. Consistent with DiR-labeled EV live imaging in CIA mice, we observed that mCherry-carrying GMSC-EVs exhibited a preference for homing to inflamed joints, while Fib-EVs did not (Figure 3D). Notably, GMSC-EVs were found to be stable and able to continuously circulate in inflamed joints after infusion. To monitor this, we conducted a time course analysis at 24 hours, 15 days, and 28 days after injecting DiR-labeled GMSC-EVs into CIA mice. The results show that a fluorescence signal was still detectable in the joints 28 days after GMSC-EV injection (Figure 3E). To summarize, our research indicates that GMSC-EVs have remarkable capabilities to migrate toward inflamed joints. Therefore, they might possess considerable promise as a therapeutic alternative for mitigating inflammatory conditions.

### Human GMSC–derived EVs exhibited a significant enrichment of miR-148a-3p.

**Figure 4:**
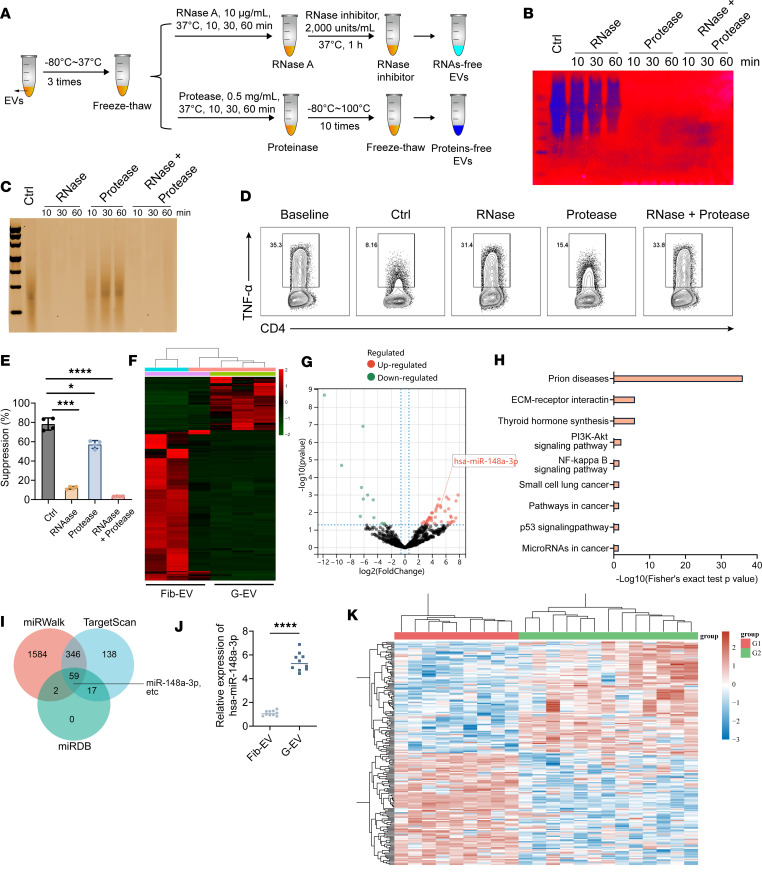
Bioinformatics analysis of the miRNA expression profile of human GMSC–derived EVs. (**A**) Flowchart illustrates the experimental procedures for removal of proteins or RNAs in GMSC-EVs. (**B**) Silver staining of polyacrylamide gel showed the protein profile GMSC-EVs upon different treatment procedures described in Methods. (**C**) The image of agarose gel showed the RNA profile GMSC-EVs upon different treatment procedures described in Methods. (**D** and **E**) In vitro suppressive assay of cytokine production. (**F**) The heatmap shows the miRNA expression profile of GMSC-EVs. (**G**) Volcano plot shows differentially expressed miRNAs. *P* < 0.05 and fold change ≥ 2 was considered statistically significant. (**H**) The pathway enrichment of the differentially expressed miRNAs was performed in online database DIANA-miRPath v.3. The *x* axis represents –log_10_(*P* value); the *y* axis represents KEGG term; *P* < 0.05 was considered statistically significant. (**I**) The predicted miRNAs to regulate IKKB from different database TargetScan, miRWalk, and miRDB. (**J**) The miR-148a-3p level in GMSC-EVs were measured by qPCR. (**K**) Heatmap of the differentially expressed genes in RA-related publicly available data set GSE56649 (13 cases of RA and 9 healthy controls). Statistical significance was assessed by 1-way ANOVA with Dunnett multiple-comparison test in **E** and by 2-tailed Student’s *t* test in **J**. Data are shown as the means ± SD from 1 of 3 independent experiments. *P < 0.05; ***P < 0.001; ****P < 0.0001.

EVs have become significant facilitators of cell-to-cell communication, transporting diverse cargo substances like proteins, lipids, mRNAs, and miRNAs to recipient cells, consequently influencing their functions (31). The objective of this research was to determine the precise elements of GMSC-EVs that are accountable for their immunoregulatory capabilities. To achieve this, we performed treatments to eliminate the proteins or RNAs present in GMSC-EVs (Figure 4A). Using these validated RNA-free and/or protein-free GMSC-EV samples, we observed that the ability of GMSC-EVs to inhibit the production of the proinflammatory cytokine TNF-α depended on the presence of RNAs within GMSC-EVs (Figure 4, B–E). The findings strongly indicate that the RNA transported by GMSC-EVs has a vital function in controlling inflammatory reactions.

To further investigate the molecular composition of GMSC-EVs, we conducted small RNA-Seq to determine their miRNA profiles (Figure 4F). Comparison with Fib-EVs revealed differential expression of 41 upregulated and 10 significantly downregulated miRNAs in GMSC-EVs (Figure 4G). Pathway enrichment analysis using DIANA-miRPath v.3 predicted the potential pathways targeted by these differentially expressed miRNAs, so as to determine the candidate pathways that can be targeted by these miRNAs (Figure 4H). In order to determine the miRNAs that regulate the IKKB/NF-κB signaling pathway, we utilized online prediction resources to generate a list of common miRNAs found in TargetScan, miRWalk, and miRDB. This was illustrated in a Venn diagram, and one of the miRNAs identified was miR-148a-3p (Figure 4I). Following this, our attention shifted to miR-148a-3p. Our biological verification aligned with the bioinformatic discoveries, demonstrating a notable abundance of miR-148a-3p in GMSC-EVs compared with Fib-EVs (Figure 4, G and J). In addition, we examined the publicly accessible data set GSE56649, which consisted of 13 cases of RA and 9 controls without any health issues, in order to discover potential genes associated with the pathophysiology of RA. Our findings indicate a notable increase in the expression of IKKB in RA compared with the controls (Figure 4K). To summarize, our results indicate that GMSC-EVs regulate the IKKB/NF-κB signaling pathway by means of miR-148a-3p, thus improving the pathology and inflammatory responses linked to inflammatory disorders.

### The immunomodulatory functions of human GMSC–derived EVs are attributed to miR-148a-3p.

**Figure 5:**
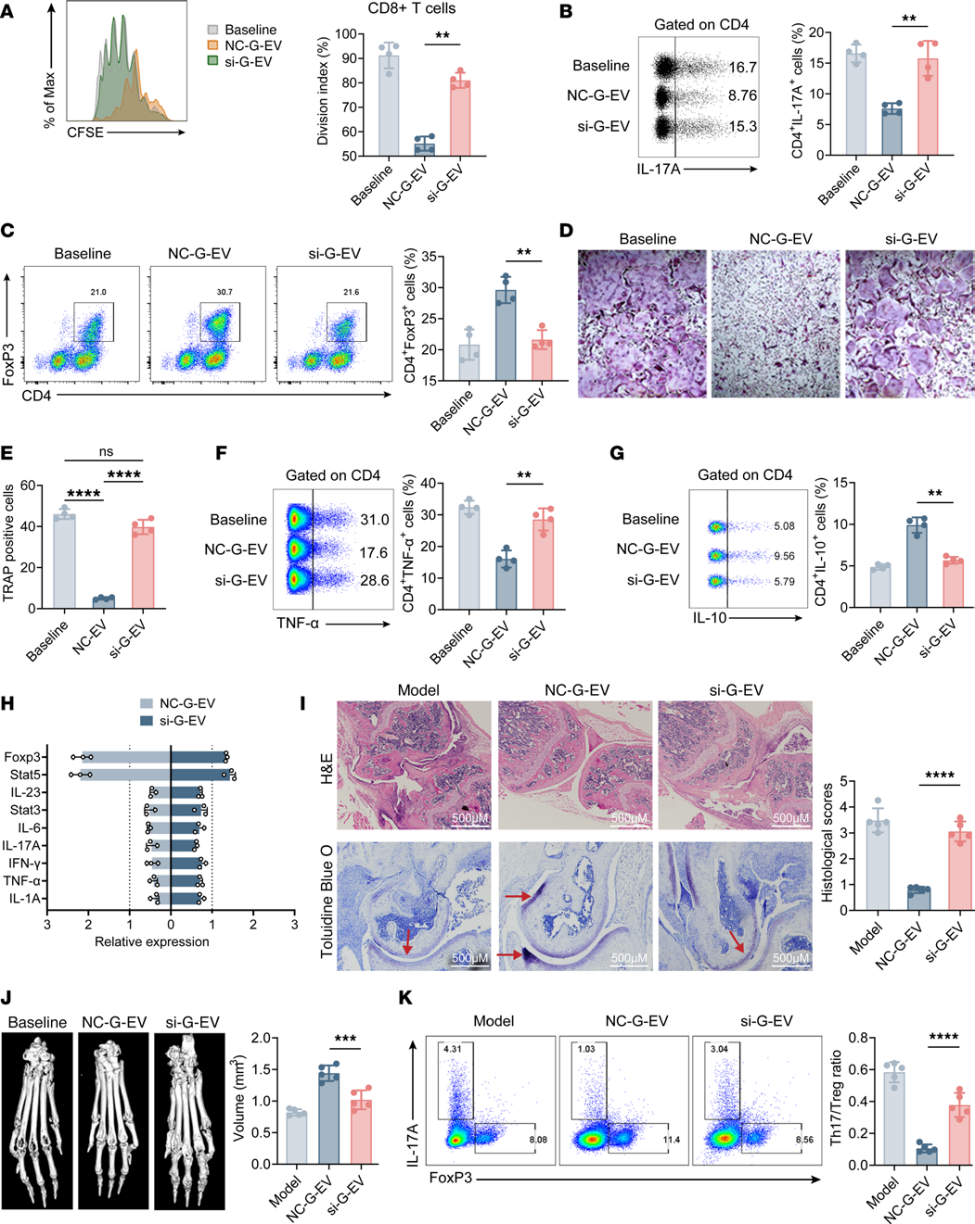
Blockage of miR-148a-3p in human GMSC–derived EVs disturbs the immunoregulatory properties. (**A**) In vitro suppressive assay of T cell proliferation. (**B** and **C**) In vitro Th17-polarizing and Treg-polarizing assays. (**D** and **E**) Representative images of osteoclast generation under different conditions. TRAP^+^ osteoclast numbers of per area under different conditions were quantified. (**F** and **G**) In vitro suppressive assay of cytokine production. (**H**) qPCR for inflammation or tolerance phenotype of CD3^+^ T cells. (**I**–**K**) CIA mice received a single type of NC-GMSC-EVs or si-GMSC-EVs at days 0, 15, and 30 after immunization, and individual analysis was acquired at the endpoint of the experiment (day 60 after immunization). Scale bars = 500 μm. (**I**) Knee joint sections were stained with H&E and toluidine blue staining, and histopathologic scores were evaluated for features of synovitis, pannus, erosion, and cartilage matrix. (**J**) Toe joint sections were imaged with μ-CT, and bone volumes of the metatarsophalangeal joints were calculated. (**K**) Intracellular staining of IL-17A and Foxp3 in dLNs was detected by flow cytometry analysis. Statistical significance was assessed by 1-way ANOVA with Dunnett multiple-comparison test in **A**–**G** and **I**–**K** and by 2-tailed Student’s *t* test in **H**. (**A**–**H**) Data are shown as the means ± SD from 1 of 3 independent experiments. In **I**–**K** data are mean ± SD, *n* = 5-8 mice. **P < 0.01; ***P < 0.001; ****P < 0.0001.

Our investigation focused on determining if GMSC-EVs modulate T cell responses via miR-148a-3p. Consistent with expectations, the inhibitory effect of miR-148a–silenced GMSC-EVs (si–G-EVs) (Supplemental Figure 4, A–C) on the proliferation of CD8^+^ T cells was less significant when compared with normal control GMSC-EVs (NC–G-EVs), which carry the normal miR-148a-3p (Figure 5A). However, si–G-EVs exhibited limited suppression of Th17 cell differentiation (Figure 5B) and osteoclast formation (Figure 5, D and E). In contrast, the activity of miR-148a-3p played a vital role in the promotion of Treg differentiation by GMSC-EVs (Figure 5C), suppression of TNF-α production (Figure 5F), and augmentation of IL-10 levels (Figure 5G). In addition, quantitative PCR (qPCR) was performed to evaluate the mRNA expression levels of various transcription factors and cytokines. The results show that miR-148a-3p plays a crucial role in the ability of GMSC-EVs to induce a tolerant T cell phenotype and inhibit the production of proinflammatory cytokines (Figure 5H).

The in vitro results, which emphasize the reliance of GMSC-EVs’ immunosuppressive role on miR-148a-3p, required further examination of their effects in vivo. In order to clarify the essential role of miR-148a-3p in the in vivo immunomodulatory function of GMSC-EVs, we performed experiments using a CIA animal model, as described earlier (Figure 2). In contrast to the beneficial therapeutic effects observed with NC–G-EVs, si–G-EVs demonstrated limited efficacy in delaying the onset of disease, reducing disease incidence (Supplemental Figure 5A), ameliorating clinical scores of arthritic pathology (Supplemental Figure 5B), and mitigating foot swelling (Supplemental Figure 5C). Moreover, si–G-EVs demonstrated limited efficacy in reducing the severity of synovial hyperplasia, damage to the cartilage (Figure 5I), erosion of the bone (Figure 5J), and regulation of the ratio of Th17/Tregs (Figure 5K). Furthermore, the administration of si–G-EVs did not effectively inhibit the synthesis of proinflammatory cytokines like TNF-α, IFN-γ, IL-17A, and IL-6. Moreover, it did not stimulate the generation of the regulatory cytokine IL-10 (Supplemental Figure 5D). Additionally, there was no effect on the levels of autoantibodies (Supplemental Figure 5E). Our results strongly endorse the requirement for miR-148a-3p in the ability of GMSC-EVs to regulate inflammatory reactions and potentially function as a treatment approach for inflammatory disorders.

### T cell response involves the direct targeting of IKKB by miR-148a-3p in GMSC-EVs.

**Figure 6:**
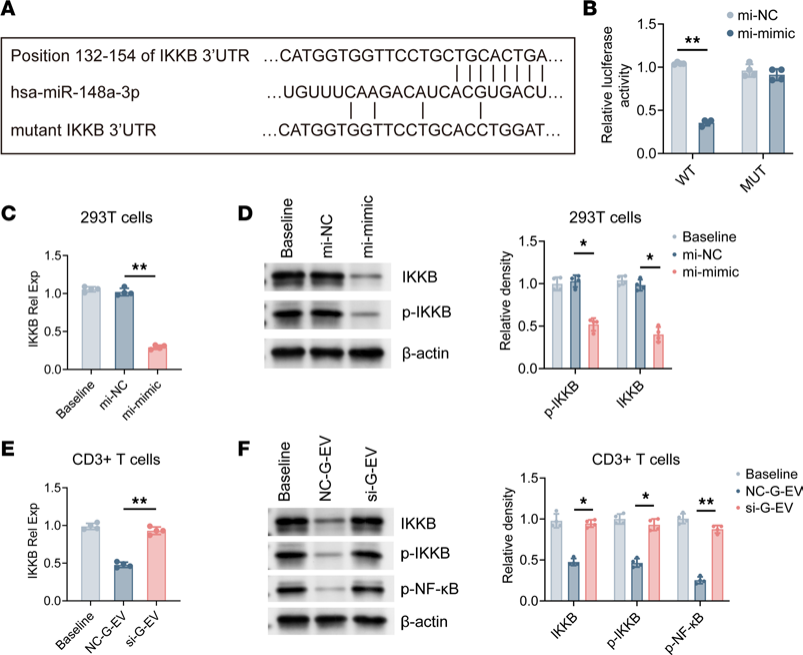
miR-148a-3p–containing human GMSC–derived EVs modulate IKKB/NF-κB signaling pathway. (**A**) Sequence alignment of miR-148a-3p and its putative target sites in the 3′ UTR of IKKB mRNA. Mutation was generated in the complementary sites for the seed region of miR-148a-3p, as indicated. (**B**) HEK293T cells were transiently cotransfected with IKKB WT or mutant 3′ UTR luciferase reporter plasmid and miR-148a-3p mimic for 48 hours, and luciferase activity was analyzed. (**C** and **D**) HEK293T cells were transiently transfected with negative control or miR-148a-3p mimic. Cells were collected at 48 hours, and the expression of IKKB or p-IKKB was detected by qPCR or Western blot respectively. (**E** and **F**) CD3^+^ T cells isolated from C57BL/6 mice were cocultured with NC-GMSC-EVs or si-GMSC-EVs under the activated condition. Cells were collected at 72 hours, and the expression of IKKB, p-IKKB, and p-NF-κB was detected by qPCR and Western blot. Statistical significance was assessed by 1-way ANOVA with Dunnett multiple-comparison test in **C**–**F** and by 2-tailed Student’s *t* test in **B**. Data are shown as the means ± SD from 1 of 3 independent experiments. *P < 0.05; **P < 0.01.

Predictions suggest that miR-148a-3p may target IKKB, an important activator of the NF-κB signaling pathway, since certain miRNAs have the ability to bind to the 3**′** UTR of IKKB mRNA and regulate its protein expression level (32). We replicated the typical and altered forms of IKKB 3′ UTR into a vector that includes a firefly luciferase reporter gene (Figure 6A). The findings of our study indicate that miR-148a-3p had a substantial effect on the expression of IKKB, which was influenced by the 3′ UTR (Figure 6B). In order to validate that miR-148a-3p directly targets IKKB at the endogenous expression level, we transfected HEK-293T cells with the miR-148a-3p mimic for 48 hours and examined the mRNA levels of IKKB. In Figure 6C, a reduction in IKKB mRNA levels was noted in cells that were subjected to treatment with the miR-148a-3p mimic. In the same way, the levels of p-IKKB and IKKB proteins were significantly reduced in cells that received the miR-148a-3p mimic treatment (Figure 6D). To confirm the essential role of miR-148a-3p in the targeting and modulation of IKKB expression in activated CD3^+^ T cells by GMSC-EVs, we examined the effect of miR-148a obtained from GMSC-EVs on IKKB in T cells. The findings of our study reveal that NC–G-EVs effectively decreased the expression of IKKB, whereas si–G-EVs did not have an effect on the levels of IKKB and NF-κB at either the mRNA or protein levels (Figure 6, E and F).

### miR-148a-3p is utilized by GMSC-EVs to improve xGvHD.

**Figure 7:**
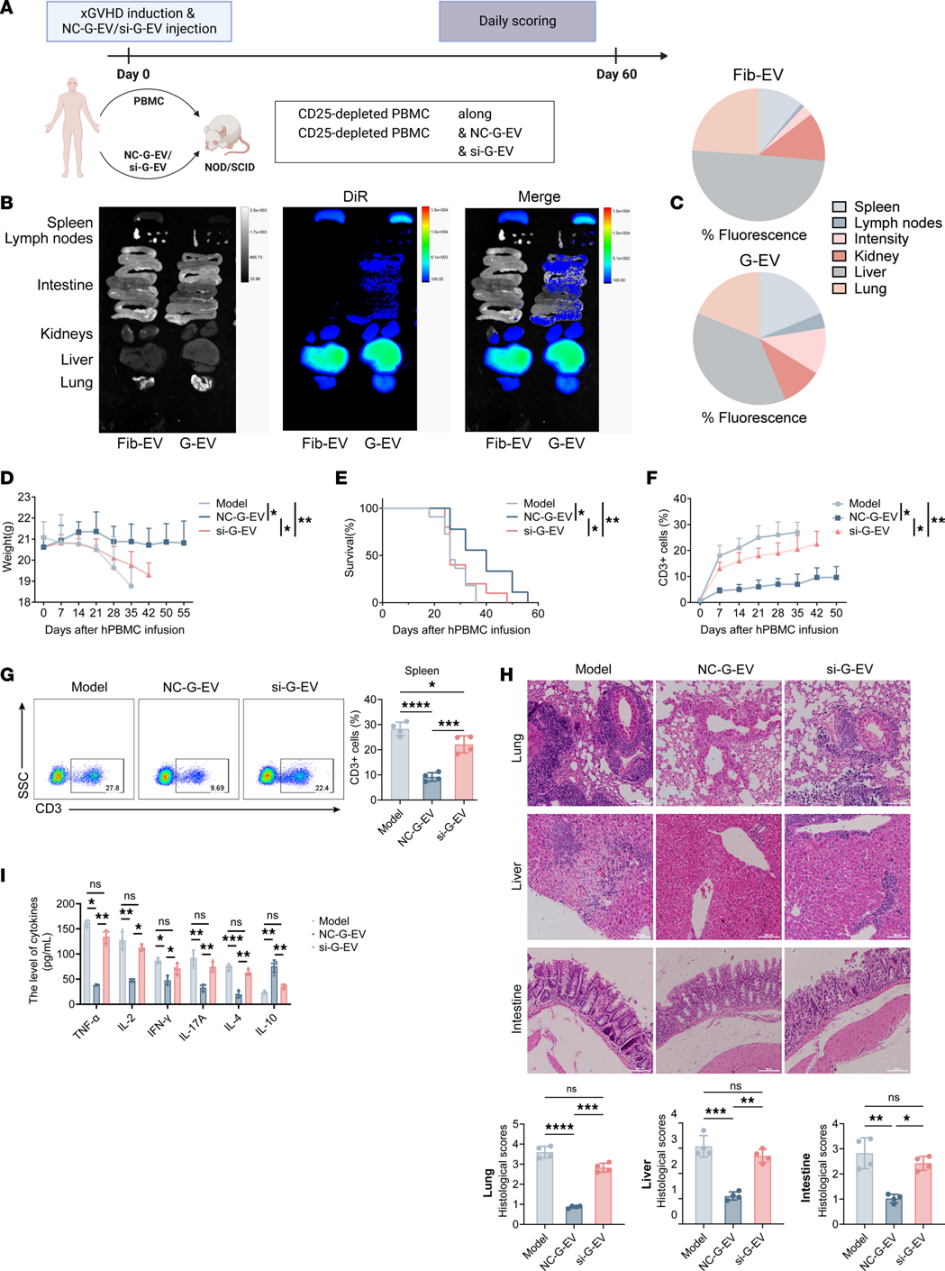
Effect of GMSC-derived EVs on xGvHD model in vivo. (**A**) Schematic experimental setup for xGvHD. (**B**) Following the administration of DiR-labeled (red) EV injections to the xGvHD mice, digital photographs and IVIS images were used to present the major organs. (**C**) Quantification of fluorescence percentage of organs for **B**. (**D**–**I**) xGvHD mice received NC-GMSC-EVs or si-GMSC-EVs at days 0, 15, and 30. The weight (**D**), survival (**E**), and human CD3^+^ T cells in peripheral blood (**F**) of xGvHD mice were monitored from day 15 to day 60. (**G**) dLNs isolated from xGvHD mice at the 50th day were used to determine the human CD3^+^ percentage by flow cytometry analysis. (**H**) Liver, lung, and intestine of NOD/SCID mice collected at the 50th day were stained with H&E, and histopathologic severity scores were determined by lymphocyte invasion. Scale bars = 500 μm. (**I**) Sera were collected from blood of NOD/SCID mice at the 50th day, and the levels of TNF-α, IL-2, IFN-γ, IL-17A, IL-4, and IL-10 were detected by ELISA. **B** and **C** show representative in vivo tracking images from 3 separate experiments. Statistical significance was assessed by 1-way ANOVA with Dunnett multiple-comparison test in **D** and **F**–**I** and by log-rank test in **E**. In **D**–**I** data are mean ± SD, *n* = 10 mice. *P < 0.05; **P < 0.01; ***P < 0.001; ****P < 0.0001.

To investigate whether the short-term rebalancing of human Treg and Th17 cells by GMSC-EVs and the crucial role of miR-148a-3p derived from GMSC-EVs in suppressing T cell immune responses in vitro have similar long-term consequences in vivo, we used a xenograft versus host disease (xGvHD) model in which human T cells are adoptively transferred into the immunodeficient mice and human cells were activated by animal antigens (Figure 7A). Initially, we used the DiR-labeling method mentioned earlier to track the dynamic distribution of GMSC-EVs in the xGvHD mice. After 24 hours of adoptive transfer, we detected DiR-labeled EVs in various organs, including the spleen, lymph nodes, intestine, kidneys, liver, and lungs. The spleen, lymph nodes, and intestine showed a higher abundance of GMSC-EVs compared with Fib-EVs, whereas both types of EVs primarily accumulated in the liver and lungs (Figure 7, B and C).

Furthermore, we assessed if GMSC-EVs could mitigate xGvHD development and investigated the involvement of miR-148a-3p in this mechanism. We observed that the xGvHD positive control mice exhibited significant mortality (refer to weight loss in Figure 7D and survival data in Figure 7E). Moreover, these mice showed an expansion of T cells, such as weekly blood phenotype (Figure 7F) and typical percentages of CD3^+^ T cells in dLNs at day 50 (Figure 7G). Nonetheless, the characteristic indications of xGvHD were significantly lessened when NC–G-EVs carrying normal miR-148a-3p were administered, whereas the administration of si–G-EVs lacking miR-148a did not yield similar outcomes. On the 50th day, we gathered different body parts from the xGvHD mice and examined the histopathological ratings of the lungs, liver, and intestines to assess the curative impacts of GMSC-EVs. According to our results, NC–G-EVs effectively decreased the histopathological scores in the various organs of the xGvHD mice. However, si–G-EVs did not successfully reduce lymphocyte infiltration or the associated pathological scores in the lungs, liver, and intestine (Figure 7H). The systemic production of proinflammatory cytokines is a notable characteristic of xGvHD. Hence, we assessed the concentrations of different cytokines in the blood samples. As anticipated, NC–G-EVs effectively suppressed the synthesis of inflammatory cytokines, including TNF-α, IL-2, IFN-γ, IL-17A, and IL-4, while enhancing the generation of IL-10. Conversely, these cytokine levels returned to untreated disease levels in the si–G-EV–treated group (Figure 7I).

### Human GMSC–derived EVs hinder the migration of RASFs and prevent them from damaging cartilage in the humanized animal model of inflammatory synovial fibroblast–mediated arthritis.

**Figure 8:**
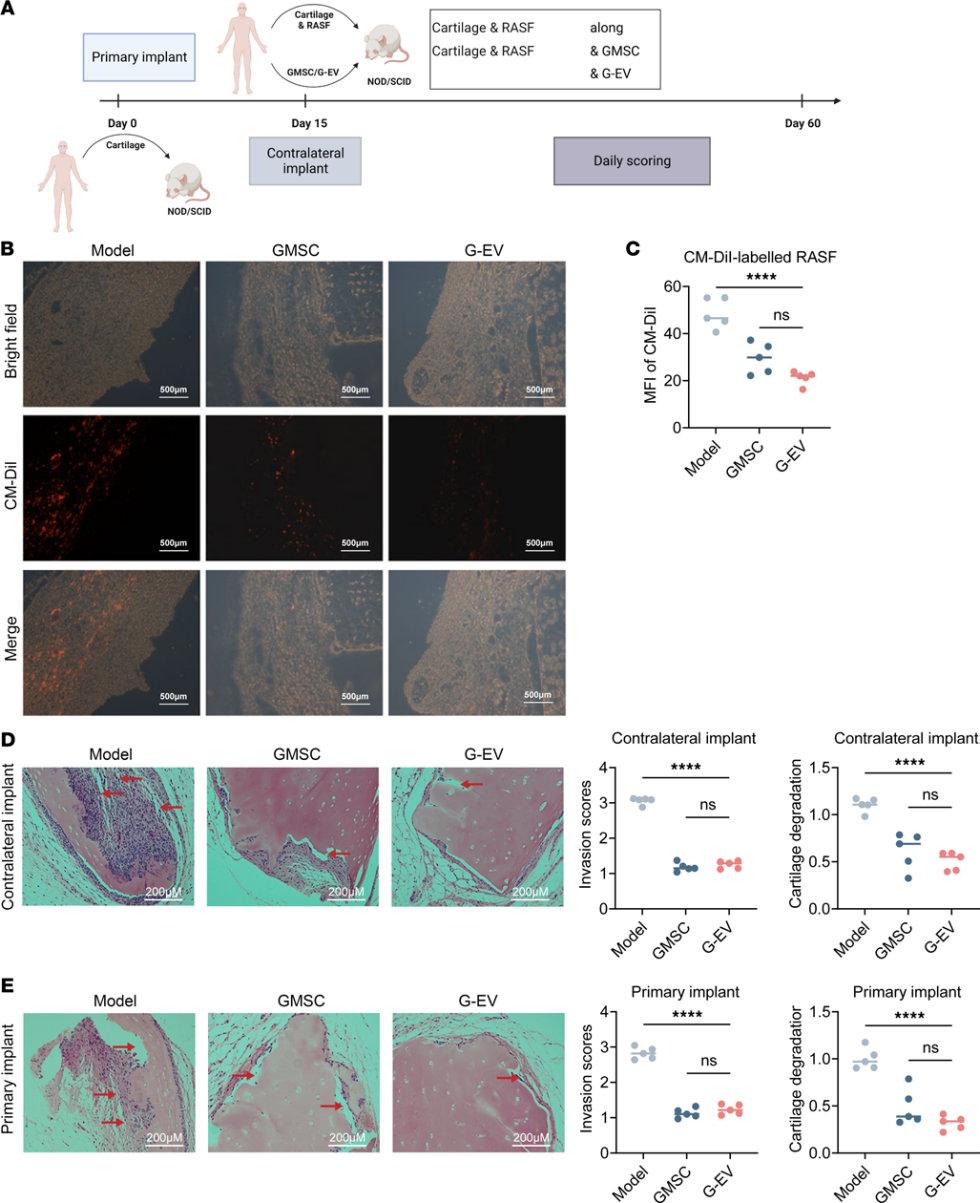
GMSC-derived EVs protect against inflamed synovial fibroblast–mediated humanized animal model. (**A**) Schematic experimental set-up for RASF-mediated humanized animal model. In the first operation, SCID mice were implanted with a cartilage-sponge complex under the left flank skin (primary implant). After 2 weeks, individual 5 × 10^5^ CM-DiI-labeled RASFs, 2 × 10^6^ GMSCs, and/or 100 μg GMSC-EVs were injected into the cartilage-sponge complex, and the implant was inserted into a s.c. space in the right flank skin (contralateral implant). (**B** and **C**) At day 60, the primarily and contralateral cartilages were collected, and the mean fluorescence intensity (MFI) of CM-DiI–labeled RASFs in primarily cartilages were quantified using ImageJ software to evaluate the invasiveness of contralateral RASFs after treatment with GMSCs or GMSC-EVs. Scale bars = 500 μm. (**D** and **E**) The contralateral and primary cartilages were collected and subjected to H&E staining to assess the invasiveness scores of inflammatory cells and the destruction of cartilages. The red arrows indicated the lesions of cartilage destruction caused by RASFs. Scale bars = 200 μm. Statistical significance was assessed by 1-way ANOVA with Dunnett multiple-comparison test in **B**–**E**. Data are mean ± SD, *n* = 5–6 mice. ****P < 0.0001.

This research project involved the creation of a humanized animal model that accurately replicates the inflammatory synovial fibroblast–mediated process observed in humans, thus effectively simulating synovial inflammation. In order to clarify if GMSC-EVs can prevent cartilage damage by controlling the aggressiveness of synovial fibroblasts, we conducted a transplantation of synovial fibroblasts from patients with RA (RASFs) into severe combined immunodeficiency (SCID) mice to induce synovitis inflammation similar to that in humans, which is mediated by RASFs (Figure 8A). To track the migration of RASFs, we initially labeled them with a red fluorescent dye called DiI, and we subsequently implanted the labeled RASFs along with healthy cartilage and therapeutic GMSCs or GMSC-EVs in contralateral sites of mice at day 15. At day 60, both the primary cartilages without direct exposure to RASFs were removed, and fluorescence microscopy revealed a significant lower fluorescence signal of RASFs in the primary cartilages of GMSC- and GMSC-EV–treated mice, indicative of the ability of both GMSCs and GMSC-EVs to suppress RASFs migration to distant sites in vivo. In contrast, the primary cartilages of GMSC-EV-treated mice exhibited a slightly reduced fluorescence signal in RASFs compared with mice treated with GMSCs (Figure 8, B and C). Moreover, the histopathological analysis with H&E staining revealed that RASFs were capable of infiltrating the cartilage and inducing significant erosion in the opposing cartilages (Figure 8D). Notably, it was observed that the main cartilage, even without direct contact with RASFs, exhibited comparable deterioration, suggesting the ability of RASFs to migrate to a remote location in living organisms (Figure 8E). Notably, both GMSCs and GMSC-EVs effectively attenuated lymphocyte infiltration and minimized cartilage destruction in both contralateral and primary cartilages (Figure 8, D and E). This observation suggests that GMSC-EVs exert direct beneficial effects not only in the local cartilage but also in cartilage that is not directly affected by RASFs. Collectively, these findings affirm that GMSC-EVs impede the invasiveness of RASFs, ultimately safeguarding against cartilage destruction in vivo.

## Discussion

MSCs are currently being investigated in many clinical trials either alone or in combination with scaffolds or biomolecules of different types. In recent years, a new group of MSCs named GMSCs has been discovered. Our team, along with other teams, has shown the powerful ability of GMSCs to modulate the immune system in various animal models of human ailments (26, 33–40). Nevertheless, the lack of a uniform MSC phenotype arises from the considerable diversity of MSCs, posing challenges in formulating standardized operational procedures (SOPs) for the clinical utilization of MSCs. EVs prepared from MSCs are highly controllable and can be made consistently without any stimulation over the parent MSCs, allowing the development of an SOP in the clinic. GMSCs have unique advantages that give them a favorable position. These advantages encompass an easily accessible source devoid of substantial trauma, swifter proliferation kinetics, and an absence of tumorigenicity risks during cell culture, as evidenced by previous investigations (41–43). These inherent benefits position GMSCs as an exemplary candidate for the generation of MSC-EVs on a mass scale.

EVs often function as transporting cargos, essentially as an intercellular shuttle to deliver biological components such as proteins and RNAs from effector cells to their target cells. MSC-EVs can modulate both innate and adaptive immunity (44). Significantly, recent inquiries have emphasized the healing effectiveness of MSC-EVs in addressing autoimmune disorders through proficiently restraining the activation of T effector cells. Consequently, MSC-EVs have garnered attention as a promising cell-free therapeutic approach (45–48). Within the context of an autoimmune disease, we utilized a CIA model to investigate the immune-modulatory capabilities of GMSC-EVs in this study. Our results unequivocally demonstrate that adaptively transferred GMSC-EVs significantly delay the onset of arthritis and improve clinical symptoms. Moreover, the development of Th17 cells, along with the simultaneous decrease in FoxP3^+^ Tregs, has been linked to the onset of RA (49, 50). In humans, the ratio of Th17 to Treg has been identified as a distinct biomarker for the progression of RA. Our current research results confirm that the transfer of GMSC-EVs effectively regulates the activation and growth of self-reactive Th17 cells, while simultaneously promoting the expansion of Tregs in mice with CIA. Our findings also reveal that GMSC-EVs reduce the levels of proinflammatory cytokines, while notably enhancing the production of IL-10. These findings align with previous studies on the immunomodulatory effects of MSCs-EVs (51–53). Collectively, our data indicate that the therapeutic efficacy of GMSC-EVs lies in their ability to tip the scales in favor of suppressing inflammatory responses while retaining immunosuppressive activity, thereby reducing the risk of developing arthritis.

Compared with conventional animal models, an anthropogenic animal model can mimic human immune disorders. The humanized animal model is the best in vivo model before clinical trials, to determine whether GMSC-EVs have the immunomodulatory efficacy of inflammation in vivo before a clinical trial. Xenogeneic guman (graft) versus mouse (host) disease (xGvHD) is established through i.v. injection of healthy peripheral blood lymphocytes into NOD/SCID mice. The development and severity of GvHD disease were determined by analyzing the survival, weight changes, organ infiltration of inflammatory cells, pathology, serum IgG, and cytology. In our recent investigation, we discovered that GMSC-EVs specifically targeted the inflamed organs and reduced the survival and progression of xGvHD, suggesting the potential translational significance of GMSC-EVs in treating inflammatory diseases mediated by human immune cells. These results underscore the potential clinical translational value of GMSC-EVs.

However, before conducting clinical trials with GMSC-EVs on patients with RA, it is crucial to utilize a humanized animal model that involves inflammation synovial cells and accurately reproduces the bone and cartilage damage features observed in RA. By utilizing this, researchers will be able to definitively establish the effectiveness of GMSC-EVs within the framework of patients with RA. The established model for studying migration and invasion of RASFs in SCID mice has previously proven to be a useful tool for preclinical research, offering significant insights and opportunities for advancements in the clinical feasibility (54, 55). In this model, RASFs could travel in SCID mice from an inflamed cartilage implant to an uninflamed site (54, 56). We have previously utilized this humanized model to explore the regulatory role of T cells in inflammatory synovitis (30, 57). During our current investigation, we made a fascinating finding that GMSC-EVs hindered the ability of RASFs to invade, ultimately offering a defense against cartilage degradation, whether or not it is seeded with RASFs. Employing this model, we have conducted a comprehensive evaluation of the protective effects exerted by GMSC-EVs and GMSCs on cartilage damage in the context of synovial inflammation. Furthermore, we have explored the capability of GMSC-EVs and GMSCs to inhibit the physiological function of human inflammatory synovial tissue.

A direct quantitative relationship between GMSCs and GMSC-EVs remains elusive, but approximately 5 million GMSCs are required to generate 100 μg of GMSC-EVs. In the inflammation synovial cell–mediated humanized animal model, 2 × 10^6^ GMSCs and 100 μg of GMSC-EVs were used. However, current results reveal that no statistically significant disparity in the impediment of RASFs invasion or the preservation of cartilage damage was observed between 2 × 10^6^ GMSCs and 10 million GMSC-generated EVs. It is important to underscore that autologous MSCs, typically functionally impaired in MSC cell therapy applications, often necessitate employment of allogeneic cells. Moreover, the quantity of MSCs that can be infused simultaneously is restricted to a predetermined threshold, thereby mandating multiple infusions to sustain or regenerate functional activity. The requirement for multiple infusions poses challenges to the autologous transplantation of cultured cells, raising the specter of uncertain differentiation and cellular distortion. Additionally, even if autologous MSCs exhibit normal functionality, autologous MSCs transplantation becomes extremely challenging in the event of an acute illness due to the time-consuming process of cell preparation and transplantation. In stark contrast, cell-free therapy utilizing MSC-derived EVs represents a distinct modality. This approach boasts minuscule immunogenicity and circumvents the obstacles associated with allogeneic transplantation rejection. MSC-EVs can be prepared proactively, endowing them with an advantageous edge in the management of emergent cases. Furthermore, administration of high-dosage EV infusions does not engender adverse effects. Consequently, the unparalleled biological attributes exhibited by GMSC-EVs confer advantages in mitigating autoimmune diseases such as RA, surpassing the capabilities of their GMSC counterparts.

In recent times, an increasing amount of proof indicates that MSC-EVs possess the ability to specifically target various organs or cell types, and it is contingent upon the presence of damaged or inflamed tissues. Conversely, MSCs could be mostly trapped in the lungs, given the size of MSCs, the lung barrier of the hosts, and the life span of MSCs in vivo after administration (58, 59). EVs exhibit a buoyant density ranging from 1.1 to 1.18 g/mL when subjected to a sucrose density gradient. Lipid rafts in their membranes are enriched with cholesterol, sphingomyelin, ceramide, and other substances (60, 61). During the formation of MVB, the EV membrane undergoes invagination, resulting in EVs acquiring the identical membrane orientation as the host cell membrane. MSC-derived EVs have the ability to readily cross any physiological barrier due to their nanoscale size, thereby enhancing their uptake efficiency by target tissues (17). A recent study reported a greater uptake specificity of MSCs-EVs for the injured kidney (62). The study successfully showcased the selective migration and circulation of GMSC-EVs to the inflamed joints in a mouse model of RA, as well as to inflamed lesions in a humanized model of xGvHD. Additionally, Shen et al. provided insights into the role of MSC-derived exosomes expressing high levels of CCR2 in the context of renal ischemia/reperfusion injury in mice. They observed a reduction in CCL2 levels, which in turn diminished the recruitment and activation of macrophages in the injured area (63). Complementing these findings, our unpublished data indicate a higher expression of CCR2, CCR7, CCR5, and CXCR5 in GMSC-EVs. These observations underscore the necessity for a more precise understanding of the mechanisms driving inflammatory homing. The potential application of this phenomenon in treating diseases characterized by physiological barriers, such as RA and multiple sclerosis, warrants further exploration.

EVs act as carriers to package proteins, lipids, mRNAs, and regulatory miRNAs derived from parent cells, and EVs transport them to target cells in order to regulate their functions (31, 64). The identification of miRNA and proteins in GMSC-EVs and their role in modulating target cells, along with the associated mechanisms, remains unexplored. It is also highly possible that either miRNAs or proteins are involved in immune modulation of MSC-EVs. miRNA, a type of small noncoding RNAs, regulates gene expression after transcription by specifically binding to the 3′ UTR region of target gene mRNA. This binding leads to destabilization of the mRNA and decreased protein expression levels of the target genes (65). MSC-EVs contain specific miRNAs that play roles in various physiological and pathological processes, including tissue regeneration, epigenetic alteration, immunomodulation, and tumorigenesis. Significantly, EVs with a membranous composition function as carriers of miRNAs, transporting operational miRNAs into specific cells. According to the report, MSC-EVs were capable of partially preventing allergic airway inflammation by delivering miR-146a-5p (66). miR-155 and miR-146a are the most extensively researched miRNAs in immune responses associated with RA. They are of particular interest in clinical settings due to their detectability in whole blood, which makes them both relevant and feasible (67). miR-146 has demonstrated its involvement in the regulation of IL-1 receptor–associated kinase 1 and 2 (IRAK1 and IRAK2), both of which play a crucial role in TLR signaling and NF-κB transcriptional activities (68, 69). High levels of the proinflammatory cytokine TNF-α in the peripheral blood are attributed to the excessive expression of miR-146a. The precise molecular mechanisms by which miR-146a operates to regulate the development and advancement of RA remain unknown.

During this research, we have made a discovery that miRNAs, instead of proteins, play a vital role as signaling mediators in GMSC-EVs to control the activities of target cells. In particular, we discovered that miR-148a-3p is abundantly present in GMSC-EVs and plays a crucial part in the immunomodulatory characteristics associated with GMSC-EVs. The initiation of the inflammatory cascade is greatly influenced by the activation of the NF-κB signaling pathway. Persistent activation of the NF-κB pathway has been implicated in various inflammatory disorders. This study shows that miR-148a-3p, present in GMSC-EVs, plays a crucial role in regulating T cells by directly inhibiting the activation of the IKKB/NF-κB signaling pathway. Blocking the expression of endogenous miR-148a-3p in GMSC-EVs led to the loss of their capacity to inhibit IKKB and NF-κB activity and to regulate the equilibrium between Th17 and Tregs.

Translational applications can greatly benefit from the numerous advantageous traits exhibited by EVs originating from MSCs. The establishment of a standardized, scalable cell culture method and robust EV isolation techniques that consistently yield immunomodulatory EVs are pivotal for developing reliable SOPs for MSC-EV–based cell-free immunotherapy in a clinical setting. Additional investigation is necessary to improve our comprehension of the healing capabilities of MSC-EVs and to uncover the molecular processes linked to their formation, variety, and specificity. Currently, MSCs are the only human cell type known to possess the ability for large-scale production of EVs, making them an attractive source for generating GMSC-EVs. GMSC-EVs harbor abundant bioactive materials within their cargo or on their surface, endowing them with significant therapeutic potential and desirable attributes as vehicles for drug delivery. Overall, our study illuminates the substantial potential of GMSC-EVs in the realm of cell-free immunotherapy, positioning them as the prime contender for extensive production of therapeutic EVs targeting RA disease. By harnessing the beneficial characteristics of GMSC-EVs, such as their reduced immunogenicity, simplified administration, and inherent ability to target inflamed tissues, GMSC-EVs emerge as a viable alternative for RA and other autoimmune diseases.

## Methods

Supplemental Methods are available online with this article.

### Sex as a biological variable.

Both male and female mice were utilized in this study, since we had previously determined that no significant differences in exist between the sexes regarding the outcomes reported in our manuscript.

### Mice.

DBA/1 J, NOD/SCID, and C57BL/6J mice were acquired from Charles River Laboratory. Mice aged between 6 and 13 weeks were employed.

### The suppression assay of T cell proliferation, differentiation. and cytokine production in vitro.

CD3^+^ T lymphocytes derived from C57BL/6J mice of the WT were isolated through the employment of the AutoMACS system, manufactured by Miltenyi Biotec. Afterward, the cells were marked with carboxyfluorescein succinimidyl ester (CFSE, 1 μM). Then, the T cells labeled with CFSE were incubated with EVs at a concentration of 20 μg/mL. In the coculture, antigen-presenting cells (APCs) treated with mitomycin C were also present, with a ratio of 1:1, along with a soluble anti-CD3 antibody at a concentration of 0.05 μg/mL. Following a period of 72 hours, the cells were gathered and subjected to flow cytometry analysis to examine the CFSE dilution in CD8^+^ and CD4^+^ T cells. The anti-CD3 antibody used in this experiment was purchased from BioLegend.

To conduct the T cell differentiation test, untainted CD4^+^CD62L^+^ T cells were extracted from the spleens of C57BL/6J mice of the WT using the AutoMACS system, ensuring a purity level exceeding 95%. The CD4 cells, which were inexperienced, were cultured using Th17- (soluble anti-CD3, 1 μg/mL, 100314; soluble anti-CD28, 1 μg/mL, 102112; rmIL-6, 20 ng/mL, P08505; rmTGF-β, 2 ng/mL, P04202; anti-IFN-γ, 5 μg/mL, 517901; anti-IL-12, 5 μg/mL, 505304; and anti-IL-4, 5 μg/mL, 504101) and Treg- (soluble anti-CD3, 1 μg/mL, 100314; soluble anti-CD28, 1 μg/mL, 102112; rmTGF-β, 2 ng/mL, P04202; and rhIL-2, 30-50 U/mL, P60568.1) inductive conditions. This was done in the presence of mitomycin C–treated APCs at a 1:1 ratio for a period of 3 days. Flow cytometry was utilized to determine the proportion of Th17 cells (CD4^+^IL-17A^+^) and Tregs (CD4^+^FoxP3^+^). BioLegend provided the anti-CD3 and anti-CD28 Abs, and R&D supplied the recombinant cytokines IL-6, IL-2, and TGF-β. Furthermore, BioLegend provided us with antibodies against IFN-γ, IL-12, and IL-4.

In order to examine the production of cytokines, we isolated splenic CD3^+^ T cells from WT C57BL/6J mice using the AutoMACS system, ensuring a purity level exceeding 95%. The cells were grown in a 48-well plate with a density of 2 million cells per well. They were then treated with soluble anti-CD3 (1 μg/mL, 100314, BioLegend) and soluble anti-CD28 (1 μg/mL, 102112, BioLegend) antibodies. Following a 72-hour incubation period, the cells were collected, and the secretion levels of TNF-α and IL-10 were examined utilizing flow cytometry.

### Establishment of CIA model.

Freund’s incomplete adjuvant (IFA) mixed 3 mg/mL heat-denatured *Mycobacterium* (Chondrex) with bovine type II collagen (C-II, 4 mg/mL) in an equal volume, resulting in an emulsion of C-II at a concentration of 3 mg/mL. As previously mentioned (27), DBA-1J mice were immunized by injecting 100 μL/mouse C-II mixture intradermally at the tail’s base. The CIA model, which is induced by collagen, is extensively employed for the examination and assessment of the pathological mechanism of potential autoimmune disorders (70). During the experiment, a single mouse was administered EVs in 100 μL of PBS at a concentration of 1 μg/μL through i.v. injection on day 0, 15, and 30. Clinical scores of arthritis features were evaluated every 2–3 days to determine arthritis incidence. Arthritis severity of every mouse was assessed and rated individually, following the previously mentioned protocols (28, 71, 72). The scores for each paw were added together to calculate a total arthritis severity score per mouse, with a maximum score of 16 for each mouse. The evaluation of each paw score was done in the following manner: 0 indicates the absence of arthritis symptoms, 1 indicates slight swelling limited to the tarsal bones or ankle joint, 2 indicates slight swelling extending from the ankle to the tarsal bones, 3 indicates moderate swelling extending from the ankle to the metatarsal joints, and 4 indicates severe swelling encompassing the ankle, foot, and digits, or limb ankylosis. The thickness of paw swelling was measured every 2–3 days. Mice were euthanized on the 60th day using CO_2_ inhalation and cervical dislocation. Histopathological examination was performed on the collected joint specimens, while μ-CT analysis was conducted on the hind limb paws. The severity of synovitis, pannus development, and bone/cartilage damage was assessed using a graded system, as outlined: grade 0 indicates the absence of inflammation, grade 1 indicates mild inflammation with synovial lining thickening but no cartilage damage, and grades 2–4 represent escalating levels of inflammatory cell infiltration and cartilage/bone destruction. The investigators, who were unaware of the experimental conditions, assessed clinical scores, arthritis occurrence, paw thickness, and histological scores.

### Histological evaluation.

Mice tissues were gathered and preserved using 10% formalin. They were then sliced into 4–7 μm sections, followed by a 30-minute exposure to a constant temperature oven set at 65°C. Afterward, the sections were soaked in xylene I for 15 minutes, followed by a 15-minute soak in xylene II. After slicing, the specimens were treated sequentially with 100% ethanol, 95% ethanol, 85% ethanol, and 75% ethanol for a duration of 5 minutes each. Subsequently, they were rinsed with flowing water for a period of 10 minutes. Sections were treated with hematoxylin aqueous solution for a duration of 5 minutes followed by eosin (H&E) staining solution for a period of 1–2 minutes. To evaluate the cartilage matrix, toluidine blue staining was conducted, while tartrate acid resistant phosphatase (TRAP) staining was carried out to measure the distribution of osteoclasts. Microscopic sections were photographed to obtain histologic images. A semiquantitative scoring system, as previously explained (73), was used to assess the histological characteristics of CIA, which encompassed synovial hyperplasia, infiltration of inflammatory cells, destruction of cartilage, and erosion of bone. Investigators who were unaware of the experimental conditions evaluated all slides.

### μ-CT analysis of bone erosion.

Hind paws were removed for CT analysis as described previously (74). In short, the scans were conducted using a 3.6 mm length that covered the entire individual paw. The scans were performed with the given parameters: a voxel size of 17.5 μm, 55 kV, 145 μA, an integration time of 200 ms, and 211 image slices. The pictures were transformed into 8-bit and imported into Mimics software (Materialise). They were then filtered using discrete Gaussian filtering with a variance of 1 and a maximum kernel width of 1. Consequently, the μ-CT system (Viva CT 40, Scanco) was used to obtain high-resolution 3D images of hind paws’ bones. Bone erosion was quantified by using volumes of interest located at the paw. Consistently, the areas of focus were aligned with the 3D longitudinal axis of the third metatarsal, and the volumes of the second through fourth metatarsal and phalangeal bones were computed.

### In vivo optical imaging.

Mice were i.v. administered with DiR-labeled or mCherry-carried EVs, equivalent to a dose of 100 μg. EVs were injected at various time intervals to examine their biodistribution in live organisms. Using the Bruker in Vivo MS FX PRO Imager (Bruker) and the IVIS 200 small animal imaging system (PerkinElmer), the mice were imaged. The excitation (Ex) filter at 700 nm and the emission (Em) filter at 780 nm (DIR) were used, along with the Em filter at 530 nm and the Em filter at 620 nm (mCherry). To establish a background measurement, the fluorescence originating from the background was measured and subsequently subtracted. The Em fluorescence was standardized to photons per second per square centimeter per steradian (p/s/cm^2^/sr). The color picture displays the arrangement of fluorescence across the creature superimposed on monochrome pictures of the mice, which were gathered simultaneously. The acquisition and analysis of images were performed using Living Image 4.0 software (PerkinElmer), as previously explained (75). The average radiance ± SD was used to express the data. Following the completion of the experiments, the mice were euthanized and the tissues (including lymph nodes, spleen, kidney, liver, lung, and intestine) were promptly imaged using the aforementioned method.

### Dual luciferase reporter gene assay.

The miR-148a-3p and IKKB putative binding sites were predicted using the biological website (http://www*.*targetscan*.*org), and their interaction was confirmed through a dual luciferase reporter gene assay. The renilla luciferase and firefly luciferase dual luciferase reporter gene in the pEZX-MT05 vector (GenePharma) had a cloned fragment of the IKKB WT and mutant (MT) 3′ UTR downstream. Next, WT or MT IKKB 3′ UTR reporter plasmids were cotransfected into HEK 293T cells with the miR-148a-3p mimic or miRNA negative control (mi-NC) using Lipofectamine 3000 (Thermo Fisher Scientific) as instructed by the manufacturer. The Dual-Luciferase Reporter Assay System (Promega) was utilized to measure luciferase activity, following the guidelines provided by the manufacturer. The luciferase activities were standardized based on the renilla luciferase activity.

### xGvHD.

After receiving 2.5 cGy total body irradiation from Rs2000 (Rad Source) (76, 77), NOD-SCID mice were i.v. administered with 20 × 10^6^ human PBMCs depleted of CD25. EVs were transfused i.v. in a volume of 100 μL PBS at a concentration of 1 μg/μL after a delay of 2–4 hours, on day 0, 15, and 30, respectively. Survival was checked daily. Weight and GvHD score were monitored every 2–3 days. Blood sample was collected once a week to test the expression of human CD3^+^ cells. Mice were euthanized on the 50th day using CO_2_ and cervical dislocation. Liver, lung and intestine isolated from mice were applied for H&E staining as described above. The assessment of the inflammation level in the liver, lung, and intestine was determined using the following criteria: 0 indicates the absence of any inflamed digits, 1 indicates 1–5 inflamed digits, 2 indicates 6–10 inflamed digits, 3 indicates 11–15 inflamed digits, and 4 indicates 16 or more inflamed digits. The investigators who were unaware of the experimental conditions assessed the histological scores. ELISA was performed on serum samples to detect the cytokines TNF-α, IFN-γ, IL-2, IL-4, IL-17, and IL-10. Flow cytometry analysis was performed using peripheral blood to determine the percentage of CD3^+^ cells in humans. Liver, lung, and intestine were applied for pathological examination.

### Inflamed synovial fibroblast–mediated humanized animal model.

On the initiation day of the animal model, a surgical procedure involving dorsal skin was performed on SCID mice. Anesthesia was induced using isoflurane, followed by a sterile incision made with surgical scissors. To minimize discomfort, bupivacaine was topically applied. Subsequently, a spongiform complex consisting of healthy donor cartilage tissue was implanted as the primary graft. Patients at The Third Affiliated Hospital of the Sun Yat-sen University and The Shanghai Jiaotong University School of Medicine were required to provide written informed consent before reaching this stage. RASFs obtained from patients with RA were cultured and stained with the CM-DiI red fluorescent labeling kit (Thermo Fisher Scientific) according to the instructions provided by the manufacturer. To label the cells, they were incubated in the CM-DiI/PBS solution at a temperature of 37°C in a dark environment for a duration of 5 minutes and were then kept at 4°C for 15 minutes. Afterward, the cells that had been labeled were rinsed with 1× PBS and then suspended in a new medium. On the 15th day, the final RASFs and a segment of healthy donor cartilage tissue encapsulated within a spongiform complex were implanted into the contralateral dorsal skin of SCID mice, serving as the contralateral implant. Either 2 × 10^6^ GMSCs in 100 μL of PBS or 100 μg of GMSC-EVs in 100 μL of PBS were injected into the contralateral spongiform complex. On the 60th day, euthanasia was performed using CO_2_ followed by cervical dislocation. The main and opposite implants (containing cartilage tissue) were extracted, and a section of the cartilage was placed in optical coherence tomography compound and frozen at –80°C. Using a Lab-Tek tissue processor (Leica), sections with a thickness of around 50 nm were acquired from the cartilage tissues. The fluorescence microscope was utilized to assess the fluorescence intensity of CM-DiI–labeled RASFs. Additionally, the excised cartilage from both contralateral and ipsilateral implants was subjected to standard H&E staining. Invasion scores and cartilage degradation were determined according to a previously reported classification system (55).

### Statistics.

The data were presented in the form of mean ± SD. Means between 2 groups were compared using a 2-tailed Student’s *t* test. One- or 2-way ANOVA with Dunnett multiple-comparison test was utilized to examine variations in the averages across several groups. Kaplan-Meier curves were used to plot survival curves and were then analyzed using log-rank tests. Statistical significance was determined by analyzing the data with GraphPad Prism Software (version 9.3), considering *P* values less than 0.05, 0.01, 0.001, and 0.0001.

### Study approval.

All patients’ informed consent was obtained. The study protocol and the use of the material was approved by the Third Hospital of Sun Yat-sen University in China and the School of Cell and Gene Therapy at the Shanghai Jiaotong University School of Medicine in China. The study was conducted following the guidelines of the Declaration of Helsinki by the World Medical Association. GMSCs were isolated and cultured from human tissues obtained from healthy donors who underwent wisdom tooth surgery at the Third Hospital at the Sun Yat-sen University in China and the School of Cell and Gene Therapy at the Shanghai Jiaotong University School of Medicine in China. The animal research was conducted following the guidelines of the animal use protocol, which received approval from the IACUC of the Third Hospital of Sun Yat-sen University and the Shanghai Jiaotong University School of Medicine. All animals were handled according to the US *Guide for the Care and Use of Laboratory Animals* and the “Principles for the Utilization and Care of Vertebrate Animals.”

### Data availability.

All data are included in the Supporting Data Values file. Any data that support the findings of this study are available from the corresponding authors upon reasonable request. The RNA-Seq data, quality control information and cluster information are available at the NCBI’s Gene Expression Omnibus data repository with the accession number GSE262961 (https://www.ncbi.nlm.nih.gov/geo/query/acc.cgi?acc=GSE262961).

## Author contributions

JC and XS performed experiment and analyzed data; JC, XS and WH wrote the manuscript; XS, YD, JD, YL, JZ, RL, DZ, WW, YX, YC, JW, WL, and XC helped in data collection; DZ and JY helped in the collection of gingival tissues; XS and YD helped in data analysis and revised the manuscript; NO, WH, YP, and QLF helped in manuscript editing; SGZ conceptualized the research, designed experiments, analyzed data, and finalized the manuscript for submission.

## Supplementary Material

Supplemental data

Unedited blot and gel images

Supporting data values

## Version 1 04/23/2024

In-Press Preview

## Version 2 05/22/2024

Electronic publication
