# Supplementary material for: miRNA-148a–containing GMSC-derived EVs modulate Treg/Th17 balance via IKKB/NF-κB pathway and treat a rheumatoid arthritis model
Source: JCI Insight. 2024 Apr 23;9(10):e177841. doi: 10.1172/jci.insight.177841 (PMC11141912; doi:10.1172/jci.insight.177841)
Supplement: Supplemental data [file jciinsight-9-177841-s149.pdf]

Supplementary Materials

MiRNA-148a-containing GMSC-derived EVs modulate Treg/Th17 balance *via* IKKB/NF-κB pathway and treat a rheumatoid arthritis model

Jingrong Chen[1,2,#], Xiaoyi Shi[2,#], Yanan Deng[1,#], Junlong Dang[1], Yan Liu[2], Jun Zhao[1], Rongzhen Liang[1], Donglan Zeng[3], Wenbin Wu[4], Yiding Xiong[1], Jia Yuan[5], Ye Chen[1,2], Julie Wang[1], Weidong Lin[1], Xiangfang Chen[6], Weishan Huang[7], Nancy Olsen[8], Yunfeng Pan[2], Qingling Fu[9,*] and Song Guo Zheng[1,*]

**Correspondence: Prof. Song Guo Zheng. 768 Zhongshan Middle Road, Shanghai, 201600, China. Tel: +86 21 6772 2300. Email: Song.Zheng@shsmu.edu.cn; Prof. Qingling Fu. 58 Zhongshan second Road, Guangzhou, Guangdong Province, 510030, China. Tel: +86 20 8733 3108. Email: fuqingl@mail.sysu.edu.cn.*

[#]*These authors contributed equally to this work.*

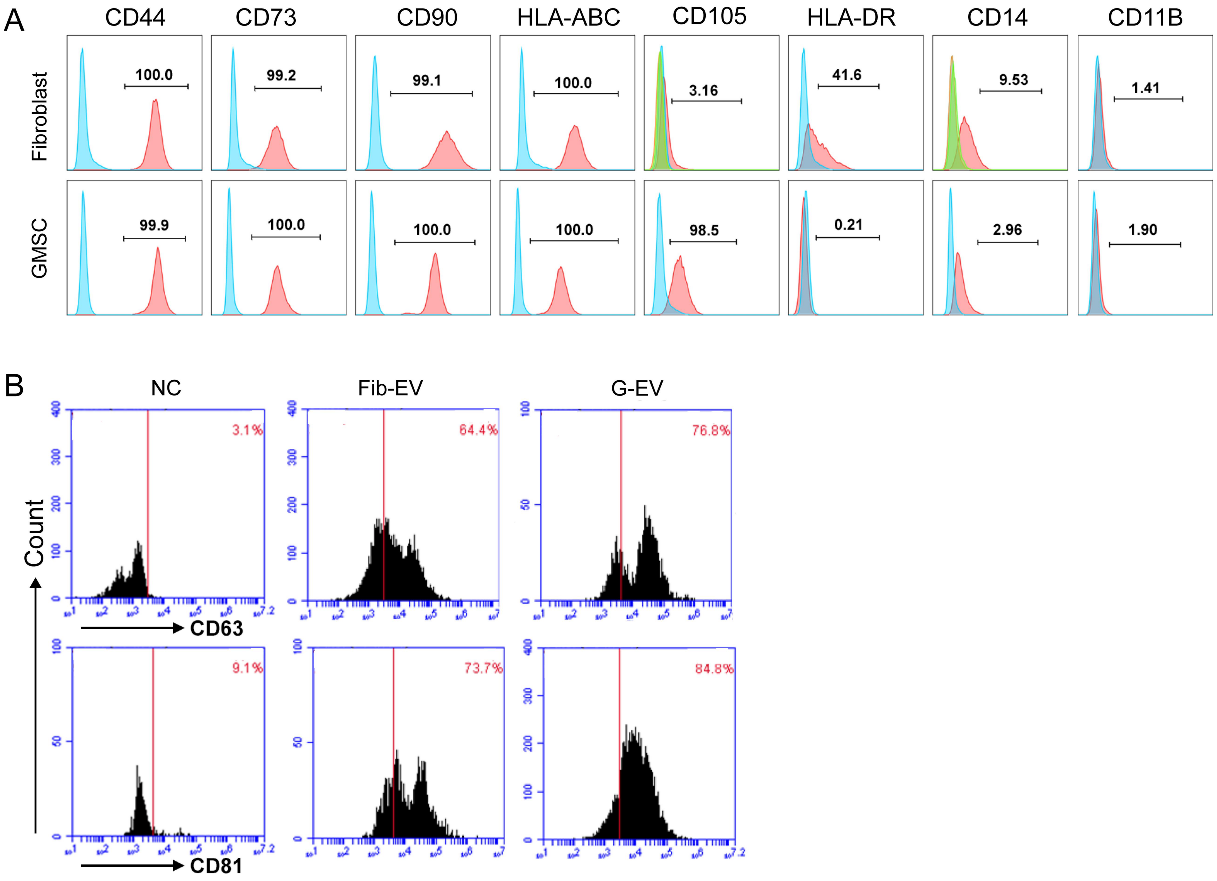

Figure S1. Characteristics of human GMSCs and G-EVs. (A) GMSC and fibroblast were obtained and phenotypic and functional characteristics were stained with human antibodies. Representative flow cytometry data showed related phenotypes of GMSC and fibroblast. **(B)** Nanoparticle trafficking analyzed the diameters and concentration of Fib-EVs and G-EVs. Data are shown as the means ± SD from one of three independent experiments.

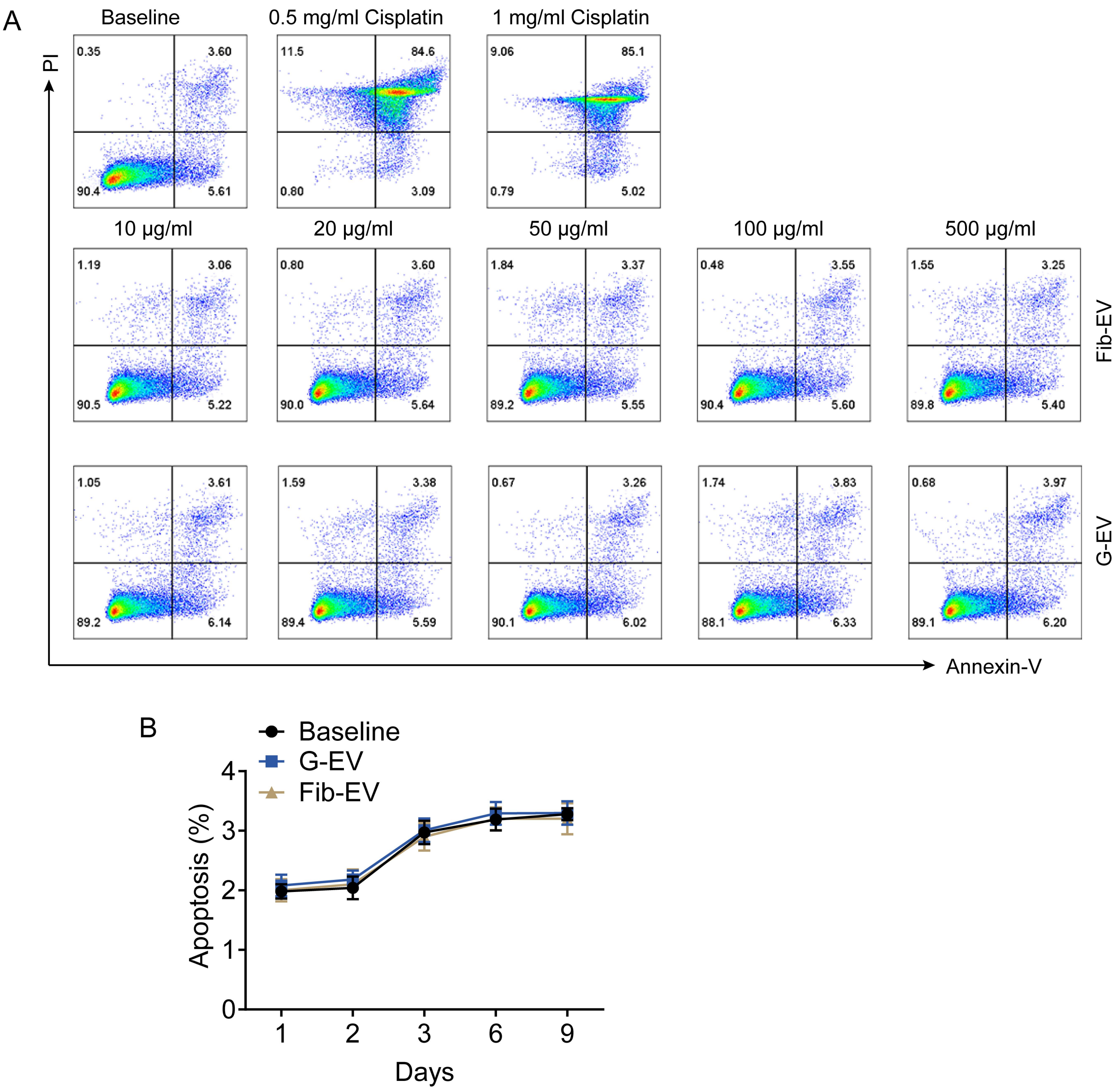

Figure S2. Staining for apoptotic markers in mouse CD3+ T cells. (A) CD3+ T cells isolated from C57BL/6 mice were co-cultured with 10, 20, 50, 100, 500 µg/mL Fib-EVs or G-EVs under stimulation of soluble anti-CD3 and soluble anti-CD28. Cells collected at 72 h were stained with Annexin-V and PI, then the percentage of apoptosis (Annexin- V+ PI+) were analyzed by FACS. **(B)** The apoptosis percentage of CD3+ T cells at 1, 2, 3, 6, and 9 days, when they were co-cultured with 20 µg/mL Fib-EVs or G-EVs. Data are shown as the means ± SD from one of three independent experiments.

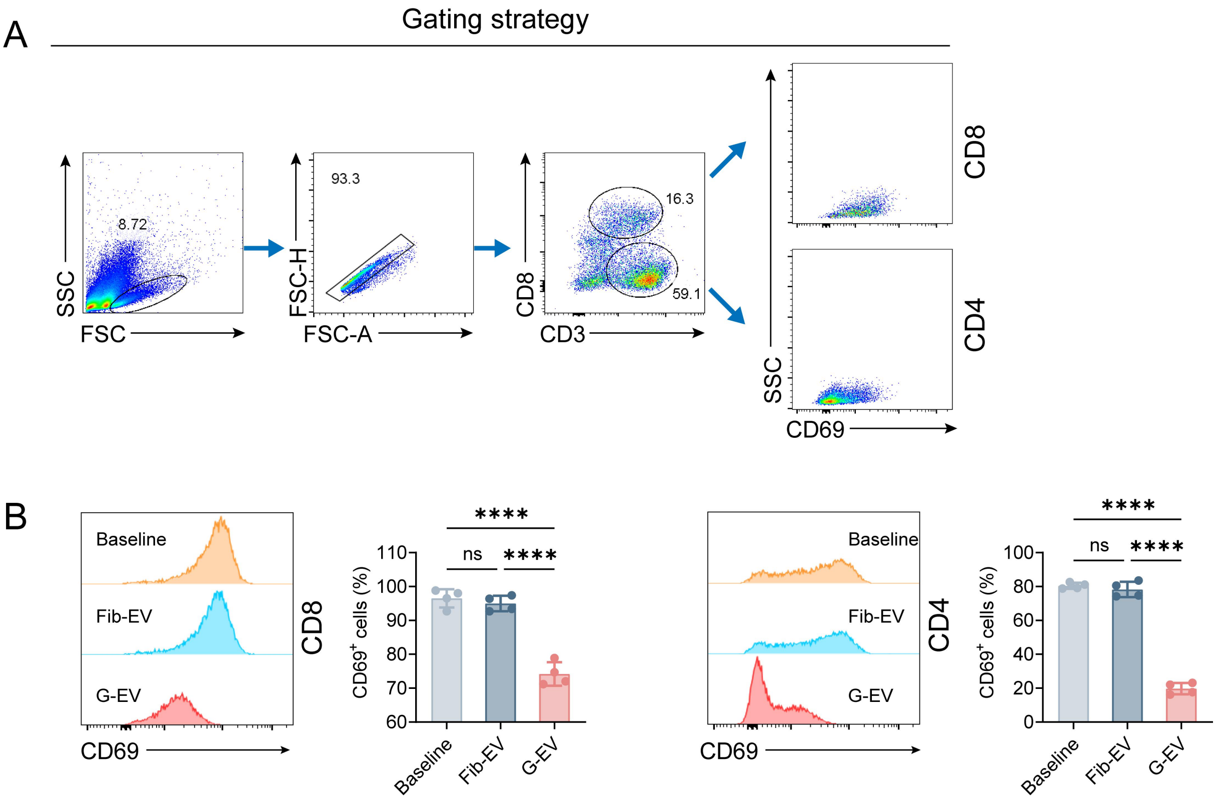

Figure S3. Human GMSC-derived EVs inhibit T-cell activation *in vitro*. (A, B) CD3+ T cells isolated from C57BL/6 mice were co-cultured with Fib-EVs or GMSC-EVs under T-cell stimulation condition. Cells were collected at 24 h, CD8+CD69+ and CD4+CD69+ cells were detected by flow cytometry analysis. Statistical significance was assessed ANOVA with Dunnett multiple comparison test in B. Data are shown as the means ± SD from one of three independent experiments. ***, $p < 0.001$; ****, $p < 0.0001$.

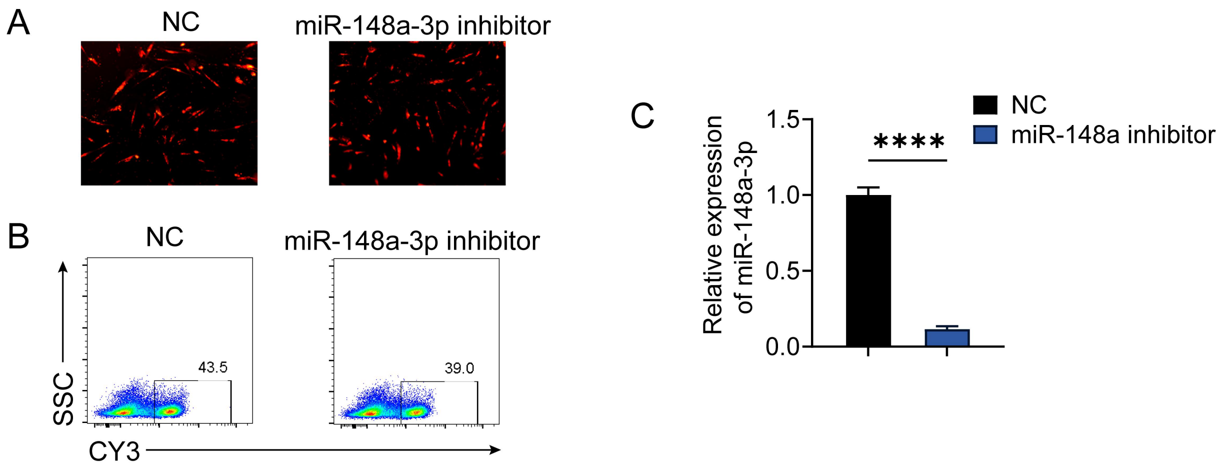

Figure S4. Transfection of miR-148a-3p inhibitor in GMSCs. (A) Fluorescence images of miR-148a-3p-CY3 transfected GMSCs. **(B)** Flow cytometer image of miR-449a-5p-CY3 inhibitor transfected GMSCs. The percentage of CY3+ was detected by flow cytometry. **(C)** The relative expression of miR-148a-3p within G-EVs after miR-148a-3p inhibitor transfection. Statistical significance was assessed with two-tailed Student t test in C. Data are shown as the means ± SD, n = 4, from one of three independent experiments. ****, $p < 0.0001$.

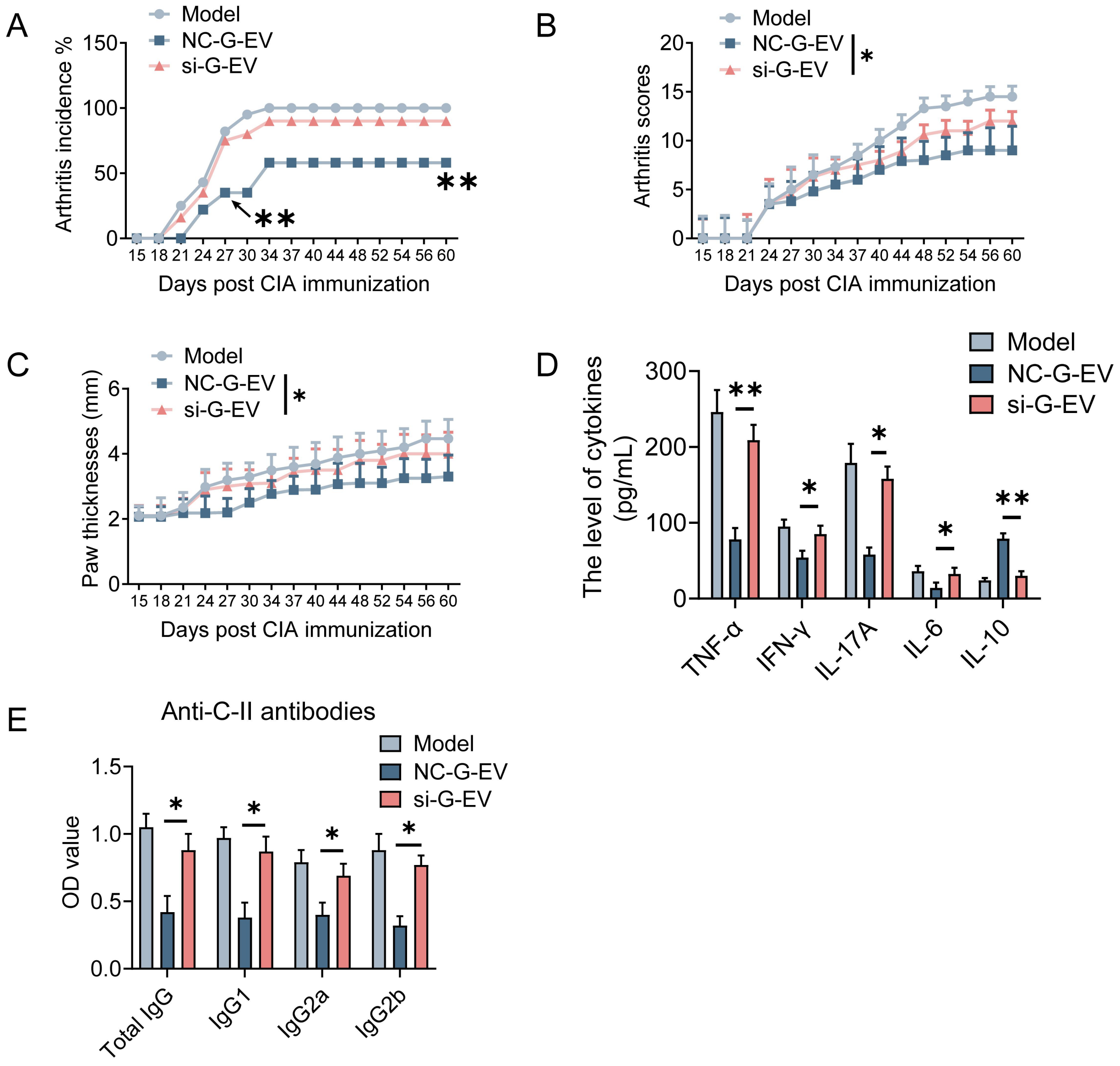

Figure S5. Blockage of miR-148a-3p disturbs the protected effects of GMSC-derived EVs on CIA mice. DBA/1 mice were used for CIA model, and mice received a single type of NC-G-EVs or si-G-EVs at day 0, 15 and 30 post immunization. The incidence of arthritis, arthritis severity scores **(B)** and paw thickness**(C)** of CIA mice were monitored from day 15 to day 60 post immunization. Serum obtained from blood of CIA mice at day 60 post immunization were used for the detection of levels of TNF-α, IFN-γ, IL-17A, IL-6, IL-10 **(D)** and anti-collagen II antibodies **(E)** by ELISA assays. Statistical significance was assessed ANOVA with Dunnett multiple comparison test in A-E. Data are mean ± SD, n = 5-8 mice. *, $p < 0.05$; **, $p < 0.01$.

Clinical data	RA
**Number (M/F)**	4 (2/2)
**Age mean, (range)**	52.5 (39-63)
**ACPA (pos/neg)**	3/1
**RF (pos/neg)**	4/1
**CRP (mg/dL), mean ± SD**	34.5 ± 8.8
**ESR (mm/h), mean ± SD**	49.6 ± 22.7
**DAS 28, mean ± SD**	4.2 ± 0.9
**SJC28, mean ± SD**	5.3 ± 4

Table S1. Clinical information of RA patients for synovial tissue samples included in this study. Antibodies to citrullinated protein antigen (ACPA), C-reactive protein (CRP), erythrocyte sedimentation rate (ESR), Disease Activity Score-28(DAS28) and swollen 28-joint count (SJC28) are shown.

Primer for Real-time RT-qPCR

Target	Primer sequence		Product
	Sense	Antisense	Size(bp)
β-actin	GTCATTCCAAATATGAGATGCGT	GCTATCACCTCCCCTGTGTG	125
IKKB	GGGGCCTGGGAAATGAAAGA	GGTCAGCCTGATTGTGCCAT	87
IL-1A	GCGTTTGAGTCAGCAAAGAAGT	CATGGAGTGGGCCATAGCTT	159
TNF-α	TGCACTTTGGAGTGATCGGC	CTCAGCTTGAGGGTTTGCTAC	146
IFN-γ	GAGTGTGGAGACCATCAAGGA	TGGACATTCAAGTCAGTTACCG	114
Stat1	GCTCGTTTGTGGTGGAAAGAC	TCTCTCATTCACATCTCTCAACT	110
IL17A	AGACCTCATTGGTGTCACTGC	CAGTCCGGGGGAAGTTCTTG	120
IL-6	CCTTCTCCACAAGCGCCTTC	GGAAGGCAGCAGGCAACA	72
Stat3	TCTGCCGGAGAAACAGTTGG	AGGTACCGTGTGTCAAGCTG	83
IL-23	CACTAGTGGGACACATGGATCT	GGTGGATCCTTTGCAAGCAG	138
Stat5	GTCACGCAGGACACAGAGAA	TGGGCAAACTGAGCTTGGAT	104
Foxp3	CACACTGCCCCTAGTCATGG	CATCCACCGTTGAGAGCTGG	116
IL-10	GGCACCCAGTCTGAGAACAG	GGCAACCCAGGTAACCCTTA	175
U6	CGCTTCGGCAGCACATATAC	TTCACGAATTTGCGTGTCAT	
hsa-miR-148a-3p	CAGTCAGTGCACTACAGAAC	3' universe	

Table S2. Primer list for RT-qPCR in this study.

Supplementary methods

Patients. The Rheumatology Department at the Third Affiliated Hospital of Sun Yat-Sen University recruited a group of 4 patients diagnosed with RA, in accordance with the criteria set by the American College of Rheumatology. Samples of synovial tissue from knee joints with active inflammation were collected from patients with RA using either Wolf 2.7 mm needle arthroscopy or ultrasound guided biopsy, both performed under local anesthesia. For patient clinical characteristics, please refer to Supplemental table 1. Patients with RA had comparable moderate to high disease activity (DAS28 4.2 ± 0.9) and biopsies had lymphocyte infiltrates and lining layer hyperplasia as scored by a clinical pathologist. The study was conducted following the guidelines of the Helsinki Declaration.

GMSCs culture and EVs preparation. Human gingiva tissues and human dermal fibroblasts were collected and prepared as previously described (1, 2). Flow cytometry was used to analyze the phenotypes of the obtained GMSCs and Fibroblasts, utilizing antibodies (Abs) specific to human CD44 (Cat.No. 397505), CD73 (Cat.No. 344004), CD90 (Cat.No. 328109), HLA-ABC (Cat.No. 311405), CD105 (Cat.No. 323217), HLADR (Cat.No. 307606), CD14 (Cat.No. 367115), and CD11B (Cat.No. 301350). These Abs were purchased from BioLegend. This study utilized GMSCs or Fibroblasts that were freshly prepared and derived from passages three to five.

GMSCs were cultured using a culture medium containing 10% fetal bovine serum (FBS) that was free of EVs. FBS was processed by centrifuging at 300 × g for 10 minutes, 3000 × g for 10 minutes, 10,000 × g for 30 minutes, and 110,000 × g for 48 hours. Afterward, it was filtered through a 0.22 μm filter (3, 4). To eliminate any potential pellet or cell debris, the culture supernatant was obtained and processed through a sequential centrifugation protocol at 300 × g for 5 minutes, followed by 3,000 × g for 10 minutes, and finally 10,000 × g for 30 minutes. Afterwards, the liquid above was subjected to ultracentrifugation at a speed of 110,000 g for 2 hours at a temperature of 4°C. The EVs were then cleansed using phosphate buffer saline (PBS) and underwent another round of ultracentrifugation at 110,000 g for 2 hours at 4 °C to remove protein aggregates. Lastly, the solution was filtered through a 0.22 μm filter (5, 6). The EVs pellet was suspended in PBS and kept at -80 °C for future investigations. The centrifugation procedures were conducted at a temperature of 4 °C.

Identification of EVs. The isolated EVs were characterized using transmission electron microscopy (TEM). In brief, 3 μl of EVs pellet was placed on formvar carbon-coated 200-mesh copper electron microscopy grids, incubated for 5 min at room temperature, and then was subjected to standard uranyl acetate staining. Prior to analysis in the transmission electron microscope (Hitachi H7500 TEM, Tokyo, Japan), the grid was rinsed with PBS three times and left to partially dry at room temperature.

The NanoSight NS300 (Malvern, UK) was utilized to conduct an examination on the distribution of EVs based on their absolute size. Particles were tracked and sized automatically using NTA, relying on Brownian motion and the diffusion coefficient. Following isolation, the EVs were thinned down in 1 mL of screened PBS (0.22 μm filter). The NTA measurement parameters included a temperature of 23. 75 ± 0.5 °C, a frame rate of 25 frames per second, and a measurement duration of 60 seconds. The detection threshold remained consistent across all the samples. Each sample underwent three counts.

EVs were incubated with aldehyde/sulfate latex beads (ThermoFisher, MA, USA) at a temperature of 4 °C overnight, using a total protein quantity of 1 μg (determined by BCA). To block any remaining reactive sites on the beads, 100 mM glycine was added. Beads coated with EVs were subjected to centrifugation at a speed of 3000 × g for a duration of 20 minutes, followed by three washes in PBS. with fluorophore-conjugated specific antibodies for CD63, and CD81 (BD LSRFortessaTM, BD Biosciences, San Jose, California, USA) for 30 minutes and data were acquired using flow cytometry EVs-coated beads were stained. Western blot analysis was performed to examine the expression of CD63 (Abcam, ab213090), TSG101 (Abcam, ab83), CD81 (Abcam, ab109201), and CD9 (Abcam, ab92726), which are markers in EVs, at a protein equivalent of 30 μg.

Fluorescent labeling of EVs and confocal microscopy. The EVs were labeled using a PKH67 green fluorescent labeling kit (ThermoFisher, MA, USA) as per the instructions provided by the manufacturer. Briefly, 100 μL EVs-PBS solution was mixed with 0.2 mL Diluent C and then 0.5 μL PKH67 dye was added and mixed for 4 min at room temperature. In order to attach surplus dye, 1 mL of 0.5% bovine serum albumin in PBS was introduced. After being subjected to centrifugation at 100,000 × g for 1 hour, the labeled EVs were washed and then suspended in PBS for the purpose of conducting uptake experiments. Next, CD3+ T cells were co-cultured with 20 μg of PKH67-labelled EVs. At the 24-hour time point, cells were gathered, then treated with CM-DiI (ThermoFisher, MA, USA) for red fluorescence staining and DAPI (ThermoFisher, MA, USA) for blue fluorescence staining after being fixed. A confocal microscope (Zeiss LSM800, Germany) was used for imaging.

Labeling EVs with DiR. In order to track EVs in vivo, EVs were suspended in PBS containing 5 μM DiR (ThermoFisher, MA, USA) and labeled with fluorescence. Following the blending process, EVs were placed in the DiR/PBS mixture for 15 minutes at room temperature without light exposure. Subsequently, they were rinsed with PBS at a centrifugal force of 120,000 g for three cycles lasting 1 hour each. The last EVs were suspended again in PBS and promptly utilized for in vivo Optical imaging (OI) test.

Generation of mCherry-carried EVs. The construction of a lentiviral expression vector, pLenti6. 3/V5-DEST, which contains the mCherry and GFP dual-fusion reporter gene, was achieved using established protocols (7, 8). Using Lipofectamine 3000 (ThermoFisher, MA, USA), the expression vector pLenti-CMV-CD63-mCherry-GFP and the packaging plasmid mix (pMD2.G and psPAX2) were co-transfected into HEK-293T cells (XY-XB-1205, ATCC). After centrifugation of the culture supernatants, the CD63-mCherry-GFP virus particles were harvested. During a 60-hour incubation period, GMSCs or Fibroblasts were exposed to the CD63-mCherry-GFP lentivirus at an MOI of 21 and 5 μg/mL polybrene for infection. The EVs were subsequently isolated following the previously described method. Afterward, the EVs labeled with mCherry were acquired and preserved at a temperature of -80 °C for additional experimental analysis.

Small RNA sequencing and bioinformatic analysis. Total RNA from EVs was isolated using miRNeasy Serum/Plasma Kit (QIAGEN) according to the protocol. The quantity and quality of total RNA were assessed using the Agilent 2100 Bioanalyzer (Applied Biosystems, Carlsbad, CA). After removing polyadenylated mRNA with oligo-dT Dynabead, RNA was concentrated through ethanol precipitation at -20 °C for one night. The NEBNext Multiplex Small RNA Library Prep Set for Illumina was utilized to prepare a small RNA library. The qPCR-based KAPA Biosystems Library Quantification kit (Kapa Biosystems, Inc., Woburn, MA) was used to determine precise measurement for sequencing purposes. Before clustering, all libraries were diluted to a concentration of 2 nM and then combined at equimolar concentrations. Sequencing of 150 bp paired-end (PE150) was conducted on the Illumina Hiseq 2500 Genome Analyzer platform in pairend mode (illumina, San Diego, CA, USA). Raw reads were quality-controlled using Fast-QC (*http://www. bioinformatics. babraham. ac. uk/projects/fastqc/*). BWA was used to map clean reads to the miRBase database (*http //www. mirbase. org/*) in order to detect familiar small RNAs. The EBSeq was used to identify miRNAs that were expressed differentially (9). The miRNAs were considered up-regulated or down-regulated if their fold change was ≥ 2 and the p-value was less than 0.05. The RNA-seq information has been submitted to the NCBI Gene Expression Omnibus (GEO) with the accession code (SRP251158). By utilizing volcano plot filtering, we successfully identified miRNAs that exhibited significant statistical differences in expression. The miRNAs were considered up-regulated or down-regulated if their fold change was ≥ 2 and the p-value was less than 0.05. Cluster 3.0 software was used to create heat maps that display genes with differential regulation. The biological pathway enrichment of the differentially expressed miRNAs was performed in online database DIANA-MirPath v.3 (*http://diana.imis.athena-innovation.gr/DianaTools/index.php?r=site/page&view=software*). The platforms used for predicting target miRNAs of IKKB were TargetScan (*http //www. targetscan. org/*), miRWalk (*http //mirwalk. umm. uni-heidelberg. de/*), and miRDB (*https://mirdb.org/*).

Elimination assay for exosomal proteins or RNAs. A comprehensive range of treatments was developed to examine the particular function of proteins or RNAs in the regulation of inflammation facilitated by GMSC-EVs. In order to isolate RNA-containing EVs while eliminating proteins, a sequential approach was followed. Initially, EVs were subjected to three freeze-thaw cycles, alternating between temperatures of -80 ˚C and 37 ˚C, with each cycle lasting for 10 minutes. Afterward, the EVs underwent treatment with protease (Sigma) at a concentration of 0.5 mg/mL and were kept at 37 ˚C for 10, 30, and 60 minutes. To deactivate the protease activity, the medium containing EVs underwent ten additional freeze-thaw cycles, this time ranging from -80 ˚C to 100 ˚C. Conversely, to obtain protein-containing EVs while removing RNAs, a similar protocol was employed. Following three freeze-thaw cycles, the EVs were subjected to RNase A (Takara) at a concentration of 10 μg/mL for 10, 30, and 60 minutes. After that, the EVs were treated with an RNase A inhibitor (Takara) at a concentration of 2,000 units/mL for 1 hour in order to counteract the effects of RNase A. For the isolation of EVs devoid of both RNAs and proteins, a combination approach was implemented. The EVs underwent treatment with RNase A for durations of 10, 30, and 60 minutes, in addition to protease, followed by a 1-hour incubation with RNase A inhibitor. Furthermore, ten freeze-thaw cycles, spanning from -80 ˚C to 100 ˚C, were also conducted.

Silver staining and agarose gel electrophoresis assessment. The GMSC-EVs underwent distinct treatment protocols as mentioned in the aforementioned methodology. Subsequently, 5× SDS loading buffer was introduced to the samples to facilitate denaturation under conditions of 5 minutes at 95 ˚C. After separation through SDS-PAGE, the resulting samples were assessed for protein elimination in EVs using silver staining. In short, the gel was treated with a mixture of 30% methanol and 10% acetic acid for 15 minutes, then sensitized for 1 minute. Subsequently, the gel was immersed in a silver reaction buffer for 5 minutes, allowing for development over a period of 2-3 minutes. The gel was then washed with stop solution, and imaging was conducted using Kodak autoradiography film (Kodak XAR film). In order to assess the elimination of RNAs in EVs, a subset of EVs that had been subjected to the aforementioned treatment procedures were separated on an agarose gel. Imaging of the separated RNAs was carried out using Kodak autoradiography film (Kodak XAR film).

Transfection of miR-148a-3p mimic and inhibitor. The authors have suggested a transfection-based approach for the purpose of modifying EVs, as reported in previous literature (10, 11). In accordance with the manufacturer's instructions, Lipofectamine 3000 (Thermo, MA, USA) was used to transiently transfected GMSCs with miRNA negative control (NC) and miR-148a-3p inhibitor, as part of the implementation of this approach. Following a period of 6 hours, culture medium was replaced with a conventional medium for an additional 72 hours of culture. Subsequently, individual culture mediums were collected to prepare EVs, utilizing the procedures described above. Lipofectamine 3000 was used once

more to transfect HEK-293T cells with miRNA negative control (NC) and miR-148a-3p mimic. Further investigation was conducted on HEK-293T cells collected 48 hours post transfection. GenePharma (Shanghai, China) synthesized the NC and miR-148a-3p inhibitor used in these experiments.

Osteoclastogenesis. CD11b+ cells from mouse bone marrow were separated using the AutoMACS system, employing a biotin anti-mouse CD11b antibody (BioLegend) and anti-biotin MicroBeads (Miltenyi Biotec) separation method, which resulted in a purity of more than 95%. Afterward, the isolated CD11b+ cells were grown in 48-well dishes using α-minimum essential medium (MEM) enriched with 10% FBS. In the culture medium, there was a presence of macrophage colony-stimulating factor (M-CSF) with a concentration of 50 ng/mL, which lasted for a period of 3 days. After that, the cells were additionally treated with RANKL (50 ng/mL, 6449-TEC-010/CF, R&D) and M-CSF (50 ng/mL, 216-MCC-025/CF, R&D) for another 6 days to promote the development of osteoclasts. In order to evaluate the degree of osteoclast development, the cells were stained with a TRAP kit (Sigma-Aldrich; 387A) following the guidelines provided by the manufacturer. Afterwards, TRAP+ cells were observed and counted using microscopy.

Quantitative Real-time PCR (qPCR) for cells. The quick RNA extraction kit (EZbioscience, USA) was used to extract total RNA from the samples, following the protocols provided by the manufacturer. Afterwards, initial-strand complementary DNAs were produced from 0.5 μg of entire RNA in a 10 μL reaction size using reverse transcriptase (5× RT Master Mix, TaKaRa, Japan). To enhance, a 1 μL portion of the reverse transcription product was utilized alongside TB Green™ Premix (TaKaRa, Tokyo, Japan). Sangon Biotech (Shenzhen, China) created and produced the particular primer sequences. The Quantstudio 5 instrument (ThermoFisher, MA, USA) was used for amplification, following a three-step thermal profile. This profile included an initial denaturation step at 95 °C for 30 s, followed by 40 cycles of denaturation at 95 °C for 5 s, and annealing/extension at 60 °C for 30s. The amplified products were then quantified by measuring cycle thresholds (CT) for each target gene and β-actin mRNA. The $2-\Delta\Delta CT$ method was implemented for quantitative analysis and statistical calculations. Sangon Biotech (Shanghai, China) synthesized the primer sequences (Supplemental table 2).

Quantitative Real-time PCR for EVs. TRIzol (ThermoFisher, MA, USA) was utilized to extract the entire RNA, and the miRNAs tail was subsequently reverse transcribed using the Mir-X miRNA First-Strand Synthesis kit (TaKaRa, Tokyo, Japan) in accordance with the instructions provided by the manufacturer. Subsequently, qPCR was conducted utilizing the Quantstudio 5 (Thermo, MA, USA). The information was examined utilizing the comparative Ct technique. To determine the miRNA levels in the cells, the data were normalized to U6 for relative comparison. Each reaction was performed three times. Supplemental table 2 contains

a list of miRNA primers that were synthesized by Sangon Biotech (located in Shanghai, China).

Western blotting. The cell lysate containing 30 μg of total protein was separated on 10% sodium dodecyl sulfate–polyacrylamide (SDS–polyacrylamide) gel, then transferred onto polyvinylidene difluoride membranes (Millipore, Bedford, USA). Following a 1.5-hour blockage in a solution containing 5% skim milk powder at ambient temperature, the subject was subsequently incubated overnight at 4 °C with primary antibodies targeting RorγT (ab207082, Abcam), p-NF-κB (#4764, Cell Signaling Technology), p-IKKB (#2697, Cell Signaling Technology), and IKKB (#8943, Cell Signaling Technology), alongside β-actin (ab8226 , Abcam) serving as the internal control. Abs were diluted in 1× TBST containing 5% BSA. Following three washes, the membrane underwent incubation with a secondary antibody conjugated with HRP at a dilution of 1:5000 for 1 hour at ambient temperature. The bands were generated utilizing a SuperSignal^{TM} West Chemiluminescent Substrate kit provided by ThermoFisher in Massachusetts, United States. The autoradiographic films were scanned (Kodak XAR film) and quantitated using Quantity One software (Bio-Rad, Hercules, CA).

Flow cytometry analysis. Fluorochrome-labeled antibodies specific for surface markers CD3, CD4, and CD8 were utilized for conducting surface staining. To evaluate the expression of FoxP3, cells were fixed and permeabilized with the FoxP3 staining buffer set (eBioscience, San Diego, CA) according to the guidelines provided by the manufacturer. In order to identify TNF-α, IL-10, and IL-17A within cells, PMA (PHORBOL 12-MYRISTATE 13-ACETATE) was used to stimulate the cells at a concentration of 50 ng/mL, along with ionomycin at a concentration of 500 ng/mL. Brefeldin A was present during the 5-hour duration. Afterwards, the cells were treated with fixative, made permeable, and then stained using protocols that had been previously published. The BD LSRFortessa™ instrument (BD Biosciences, San Jose, California, USA) was utilized to obtain flow cytometry data, while data analysis and visualization were performed using FlowJo 10. 6. 2 software (Tree Star, Ashland, OR).

Enzyme-linked immunosorbent assay (ELISA). Blood samples were collected from the retro-orbital sinus using Eppendorf tubes. Blood samples (without a blood thinner) were stored at ambient temperature for 30 minutes, then spun at 12,000 g for 15 minutes. Samples were gathered and preserved at a temperature of -80 °C. ELISA assays (Bioo scientific, USA) were used to detect the levels of TNF-α, IL-2, INF-γ, IL-6, IL-4, IL-17A, IL-10, and IgG, IgG1, IgG2a, IgG2b, following the manufacturer's instructions. The manufacturer provided standards were used to detect samples in triplicate and analyze for any notable variations among groups.

REFERENCES

1. Zhang X, et al. A protocol for isolation and culture of mesenchymal stem cells from human gingival tissue. *Am J Clin Exp Immunol*. 2019;8:21-26.
2. Zhao J, et al. Human gingiva tissue-derived MSC ameliorates immune-mediated bone marrow failure of aplastic anemia via suppression of Th1 and Th17 cells and enhancement of CD4+Foxp3+ regulatory T cells differentiation. *Am J Transl Res*. 2019;11:7627-7643.
3. Feng Y, et al. Heat shock improves Sca-1+ stem cell survival and directs ischemic cardiomyocytes toward a prosurvival phenotype via exosomal transfer: a critical role for HSF1/miR-34a/HSP70 pathway. *Stem Cells*. Feb 2014;32:462-72.
4. Lai P, et al. A potent immunomodulatory role of exosomes derived from mesenchymal stromal cells in preventing cGVHD. *J Hematol Oncol*. Dec 7 2018;11:135.
5. Théry C, et al. Isolation and characterization of exosomes from cell culture supernatants and biological fluids. *Curr Protoc Cell Biol*. Apr 2006;Chapter 3:Unit 3.22.
6. Gardiner C, et al. Techniques used for the isolation and characterization of extracellular vesicles: results of a worldwide survey. *J Extracell Vesicles*. 2016;5:32945.
7. Maida Y, et al. Exosomal transfer of functional small RNAs mediates cancer-stroma communication in human endometrium. *Cancer Med*. Feb 2016;5:304-14.
8. Yoshimura A, et al. The Sox2 promoter-driven CD63-GFP transgenic rat model allows tracking of neural stem cell-derived extracellular vesicles. *Dis Model Mech*. Jan 30 2018;11:
9. Leng N, et al. EBSeq: an empirical Bayes hierarchical model for inference in RNA-seq experiments. *Bioinformatics*. Apr 15 2013;29:1035-43.
10. Ye S, et al. Exosomal miR-24-3p impedes T-cell function by targeting FGF11 and serves as a potential prognostic biomarker for nasopharyngeal carcinoma. *The Journal of pathology*. 2016;240:329-340.
11. Fang S-B, et al. Small extracellular vesicles derived from human mesenchymal stromal cells prevent group 2 innate lymphoid cell-dominant allergic airway inflammation through delivery of miR-146a-5p.
