# Supplementary figures and images for: miRNA-148a–containing GMSC-derived EVs modulate Treg/Th17 balance via IKKB/NF-κB pathway and treat a rheumatoid arthritis model
Source: JCI Insight. 2024 Apr 23;9(10):e177841. doi: 10.1172/jci.insight.177841 (PMC11141912; doi:10.1172/jci.insight.177841)

Figure 2J

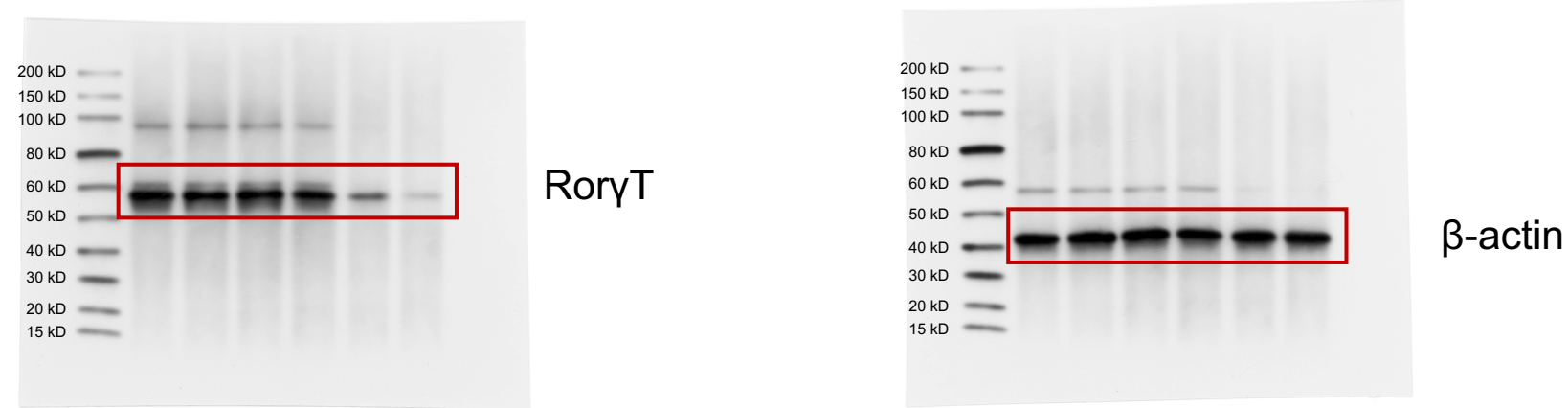

Figure 6D

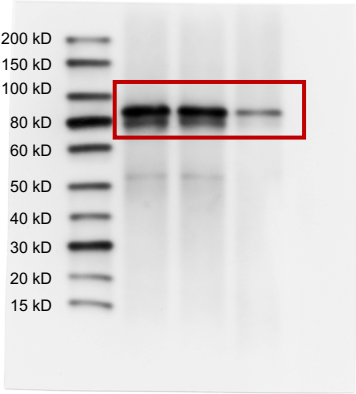

IKKB

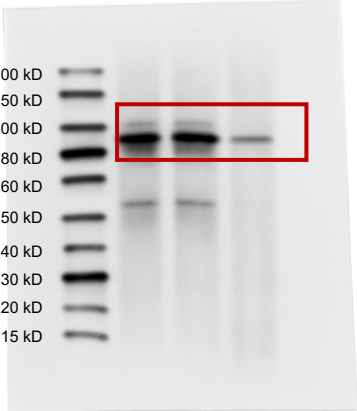

p-IKKB

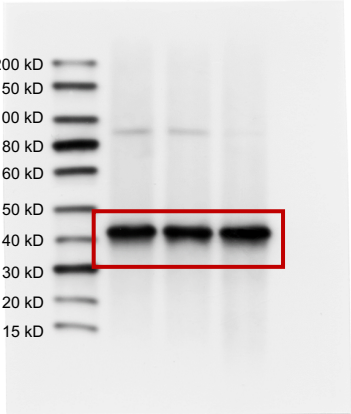

$\beta$ -actin

Figure 6F

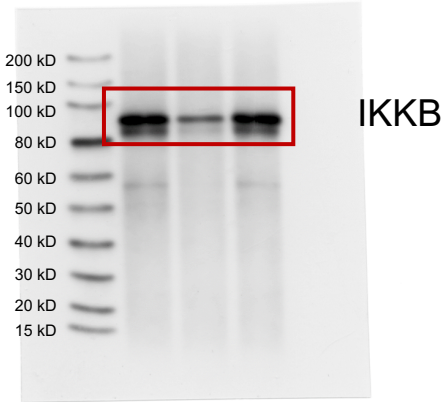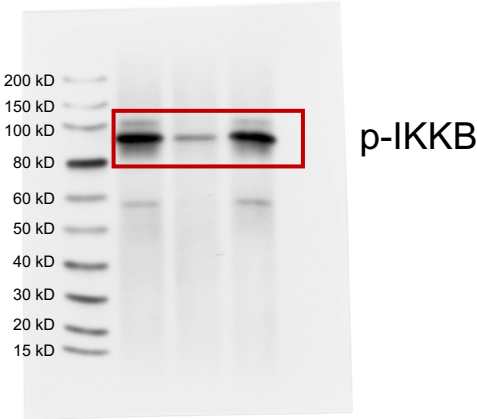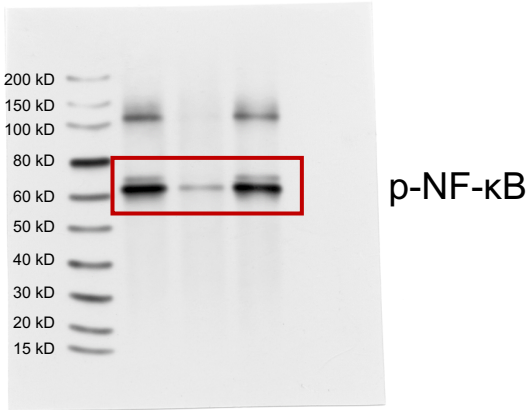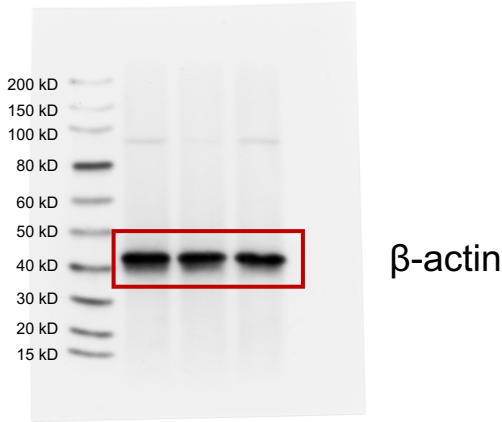

Supplement: Unedited blot and gel images [file jciinsight-9-177841-s150.pdf]
